# *De novo* transcriptome assembly of drought tolerant CAM plants, *Agave deserti* and *Agave tequilana*

**DOI:** 10.1186/1471-2164-14-563

**Published:** 2013-08-19

**Authors:** Stephen M Gross, Jeffrey A Martin, June Simpson, María Jazmín Abraham-Juarez, Zhong Wang, Axel Visel

**Affiliations:** 1 DOE Joint Genome Institute, Walnut Creek, CA, USA; 2 Genomics Division, Lawrence Berkeley National Laboratory, One Cyclotron Road, Berkeley, CA, 94720, USA; 3 Department of Genetic Engineering, CINVESTAV, Irapuato, Guanajuato, Mexico

**Keywords:** RNA-seq, Bioenergy, Crassulacean acid metabolism, *de novo* transcriptome assembly, Q420 Alternative energy sources

## Abstract

**Background:**

Agaves are succulent monocotyledonous plants native to xeric environments of North America. Because of their adaptations to their environment, including crassulacean acid metabolism (CAM, a water-efficient form of photosynthesis), and existing technologies for ethanol production, agaves have gained attention both as potential lignocellulosic bioenergy feedstocks and models for exploring plant responses to abiotic stress. However, the lack of comprehensive *Agave* sequence datasets limits the scope of investigations into the molecular-genetic basis of *Agave* traits.

**Results:**

Here, we present comprehensive, high quality *de novo* transcriptome assemblies of two *Agave* species, *A. tequilana* and *A. deserti,* built from short-read RNA-seq data. Our analyses support completeness and accuracy of the *de novo* transcriptome assemblies, with each species having a minimum of approximately 35,000 protein-coding genes. Comparison of agave proteomes to those of additional plant species identifies biological functions of gene families displaying sequence divergence in agave species. Additionally, a focus on the transcriptomics of the *A. deserti* juvenile leaf confirms evolutionary conservation of monocotyledonous leaf physiology and development along the proximal-distal axis.

**Conclusions:**

Our work presents a comprehensive transcriptome resource for two *Agave* species and provides insight into their biology and physiology. These resources are a foundation for further investigation of agave biology and their improvement for bioenergy development.

## Background

The lack of genomic and transcriptomic sequence information for agaves, succulent plants native to the arid regions of North America, limits molecular investigation of their adaptations to the abiotic stresses of xeric environments. Agaves are remarkably resistant to heat and drought stress as they employ crassulacean acid metabolism (CAM)—a water-efficient form of photosynthesis in which the uptake of CO_2_ into plant tissues through stomata and the fixation of CO_2_ into organic molecules is temporally separated [1]. CAM plants have high water use efficiency, 4–2X more efficient in water use efficiency than plants employing C3 and C4 photosynthesis [2]. Moreover, an increased CO_2_ concentration within CAM plant cells increases the efficiency of carbon fixation by Rubisco [2]. Agaves exhibit equally important morphological adaptations to xeric environments that further increase their drought and heat resistance [3]. Specialized leaves [4,5], cuticles [6-8], and roots [9,10] further protect agaves from thermal damage and prolonged drought. Agaves thus offer an opportunity to study broad-spectrum heat and drought resistance not necessarily present in all CAM plants, and provide an important model for creating applied solutions to agricultural challenges associated with climate change [1,11]. Because of adaptations to arid environments [5,12], agaves have also recently been proposed as a lignocellulosic bioenergy feedstock suitable for marginal land [13,14].

**Figure 1:**
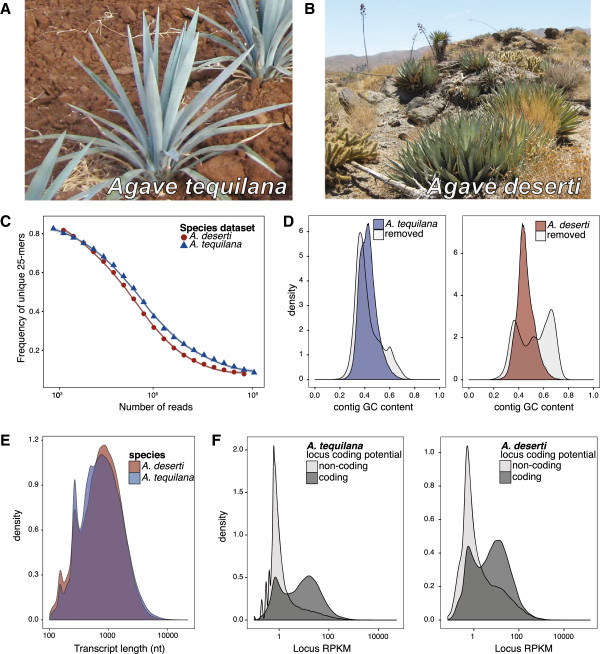
***Agave tequilana*, *A. deserti*, and an overview of their respective transcriptomes. (A)** Cultivated *A. tequilana* in Jalisco, Mexico. **(B)***A. deserti* (foreground) in Riverside County, California, USA. **(C)** Plot of the fraction of unique 25-mers over indicated read depth (log_10_ scale). **(D)** Density plot of GC content of agave transcript contigs vs. contigs from contamination and commensal organisms. **(E)** Density plots of *A. deserti* and *A. tequilana* contig lengths. Note log_10_ scale. Peaks at 150 and 250 nt represent single reads or paired-end reads, respectively, that were not assembled into larger contigs (Methods). **(F)** Density plot of locus RPKM values for coding (dark shading) and non-coding (light shading) loci.

To date, the ecology and physiology of two *Agave* species, *A. tequilana* (Figure 1A) and *A. deserti* (Figure 1B), have been studied most extensively. *Agave tequilana Weber* var*. azul*, colloquially known as the blue agave, is cultivated in western Mexico for the production of the distilled spirit tequila [15]. *A. tequilana* is of both cultural [15,16] and economic importance to Mexico, representing $1.7 billion in annual revenue within the United States alone [17]. Because of its productivity, established agricultural practices, and ethanol conversion technologies, *A. tequilana* and its close relatives represent some of the most promising *Agave* species for bioenergy [18]. *Agave deserti,* subject of numerous ecological and physiological studies [reviewed in [19], is native to the Sonoran Desert regions of the Southwestern United States and Northwestern Mexico [5] and grows within elevation ranges that experience both hot, dry summers and occasional freezing temperatures in winter [20,21]. Adapted to the conditions of its native habitat, *A. deserti* displays exceptional drought and temperature tolerance. Mature *A. deserti* plants survive up to a year without rainfall [4,22], and, in side-by-side comparisons with 14 other *Agave* species, *A. deserti* displays the largest range of thermotolerance, surviving a temperature range of 77.5°C (-16.1°C to 61.4°C) [23]. While *A. deserti* is comparatively smaller and slower-growing than *A. tequilana*, it provides a valuable model to study molecular and physiological mechanisms of plant drought and heat resistance [19,24,25].

Agaves have large genomes, estimated to be around 4 Gbp [26] with a significant amount of gene duplication due to paleopolyploidy [27] and a high number of repetitive elements [28], presenting significant challenges for genome assembly. To provide a comprehensive and accurate foundation for molecular studies of agaves, herein we present reference transcriptome datasets of *A. tequilana* and *A. deserti*, assembled from deep RNA-seq data. Cross-species comparisons demonstrate high depth and accuracy of the *Agave de novo* assemblies. Comparative transcriptome profiling provides insights into the molecular and physiological functions along the proximal-distal axis of the *A. deserti* leaf, and demonstrates broad conservation of leaf development and function across monocotyledonous plants. These reference transcriptomes provide resources for further molecular investigations of the *Agave* genus to enable their use as models for plant adaptations to abiotic stress, and improve agaves for applied bioenergy technologies.

## Results

### Deep sequencing of *Agave* tissues captures the majority of *Agave* transcripts

Both *A. tequilana* and *A. deserti* spend the majority of their 5–10 year lifespan as vegetative rosettes (Figure 1A, 1B) before a single flowering event followed by rapid senescence [5]. mRNA was harvested from various *Agave* tissues (Additional file 1: Table S1, Figure S1), and strand-specific cDNA sequencing libraries of specific insert sizes were prepared for Illumina sequencing (Methods). In total, we sequenced 978 million *A. tequilana* and 615 million *A. deserti* RNA fragments using 150 nucleotide paired-end reads (Additional file 1: Tables S2 and S3). To assess coverage of the agave transcriptomes, we plotted the frequency of observing a new unique 25-mer sequence over an increasing number of randomly sampled reads. In both data sets, the 25-mer discovery frequency decreases as sequencing depth increases, and asymptotically levels off at approximately 0.08 (8%) (Figure 1C). While complimentary datasets, such as completed genomes, will be required to conclusively determine transcriptome coverage, these observations suggest the sequencing depth was sufficient to sample the majority of sequence diversity in agave tissues. Reads from the two *Agave* datasets were separately assembled into contigs by the *de novo* transcriptome assembly pipeline Rnnotator [29]. Resulting contigs were grouped by sequence similarity into genetic loci to account for alternative splicing and reduce redundancy in downstream analyses (Methods, Additional file 1: Table S4).

To eliminate contigs derived from commensal organisms, lab contaminants, and artifacts resulting from incorrect assembly [30,31], contigs of non-plant origin were removed (Methods). Analysis of GC content of contigs from the two agave species and contaminating contigs indicates filtering produces high confidence *Agave* transcriptomes largely free of contamination (Figure 1D). Resulting *Agave* transcriptome details are summarized in Table 1 (for additional details, Additional file 1: Tables S4–S6). Assembled contigs are of similar length in both species (Figure 1E). Both agaves encode nearly identical numbers of high-confidence proteins (~35,000 each, Table 1). Transcripts from non-coding loci tend to be less abundant than transcripts from protein-coding loci (Figure 1F) (Wilcoxon rank sum test *p*-value < 0.05).

**Table 1:** **Summary of the *A. tequilana *and *A. deserti *transcriptome assemblies**

**Species**	Total sequencing*	**No. of *Agave *loci**	**No. *Agave *contigs**	**N50 length**	**Sum length of *Agave *contigs**	**No. of protein-coding loci**
*A. tequilana*	293.5 Gbp	139,525	204,530	1387 bp	204.9 Mbp	34,870
*A. deser*	184.7 Gbp	88,718	128,869	1323 bp	125.0 Mbp	35,086

Additional details on the depth of Illumina sequencing are in Additional file 1: Tables S2–S6.

*Sequence data from unusable reads where the Illumina TruSeq index could not be deciphered are not included.

### Sequence comparisons indicate high accuracy and depth of the *A. tequilana de novo* assembly

**Figure 2:**
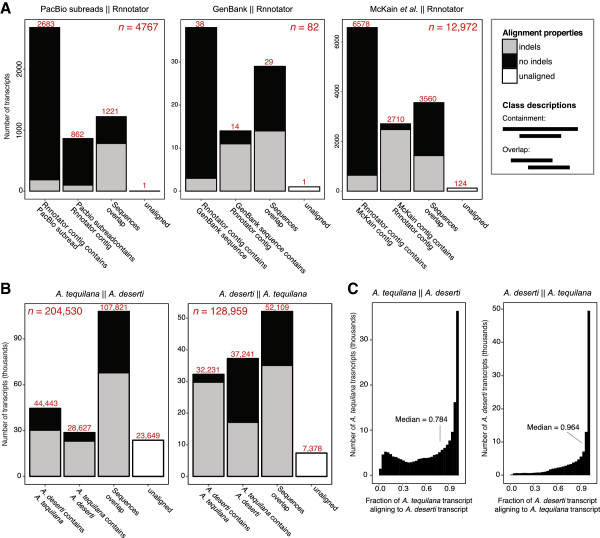
**Comparison of the *A. tequilana *Rnnotator *de novo *assembly to additional datasets and to the *A. deserti de novo *assembly. (A)** Comparison of the *Agave tequilana* Rnnotator assembly to Pacific Biosciences cDNA sequencing, *A. tequilana* sequences in GenBank, and an alternative *de novo A. tequilana* assembly built by McKain *et al.*, 2012 [27]. See text for details. **(B)** Comparisons between the *A. tequilana* and *A. deserti* assemblies. Inset–key for alignment properties. Total number of sequences compared to *A. tequilana* Rnnotator contigs (*n*) is noted in each bar chart (*n*), and number of sequences in each alignment class is noted above bar. **(C)** Histograms of the fractional alignment lengths between *A. deserti* and *A. tequilana*. Symbol || delimits query dataset from subject dataset.

To examine the accuracy of transcript assembly, we complemented our deep short-read sequencing with smaller-scale long-read single-molecule (Pacific Biosciences) sequencing of *A. tequilana* cDNAs [32] (Methods) (Figure 2A). Error-corrected, high quality subreads (N50 = 450 bp, Additional file 1: Figure S2) (Methods) were aligned to the Rnnotator *de novo* assembly (Figure 2A). We observed that 4,766 of 4,767 subreads are represented in the short-read based *de novo Agave* transcriptome assembly. We also compared the *A. tequilana* Rnnotator assembly to all 82 *A. tequilana* nucleotide sequences available from GenBank and observed that 81 (98.8%) are represented in our dataset (Figure 2A). Comparison of our *A. tequilana* Rnnotator assembly to a set of 12,972 transcripts assembled from low-depth RNA sequencing by McKain *et al.*[27] (approximately 3 Gbp, compared to 293 Gbp in the present study) (Figure 2A) reveals 12,848 of the 12,972 McKain *et al.* transcripts (99.0%) are represented in our transcriptome assembly and 8,298 transcript contigs (64.0%) align with no insertions or deletions. Of transcript contigs aligning between the two *de novo* assemblies, 6,578 (50.7%) are longer in the Rnnotator *de novo* assembly. Taken together, these comparisons further support the accuracy and near-completeness of our reference transcriptome dataset.

### *Agave deserti* and *A. tequilana* transcriptomes show high sequence identity

The transcriptomes of *A. deserti* and *A. tequilana* were compared using reciprocal BLAT analyses [33] using a minimum sequence identity threshold of 90% (Figure 2B). A significant portion of each agave transcriptome aligns to its counterpart, with 94.3% of *A. deserti* transcripts aligning to *A. tequilana*, and 88.44% of *A. tequilana* transcripts aligning to *A. deserti*. Transcripts aligning between the two *Agave* species also show a significant similarity in length and long regions of sequence alignment (Figure 2C).

### Clustering of *Agave* protein families further support *de novo* transcriptome completeness

**Figure 3:**
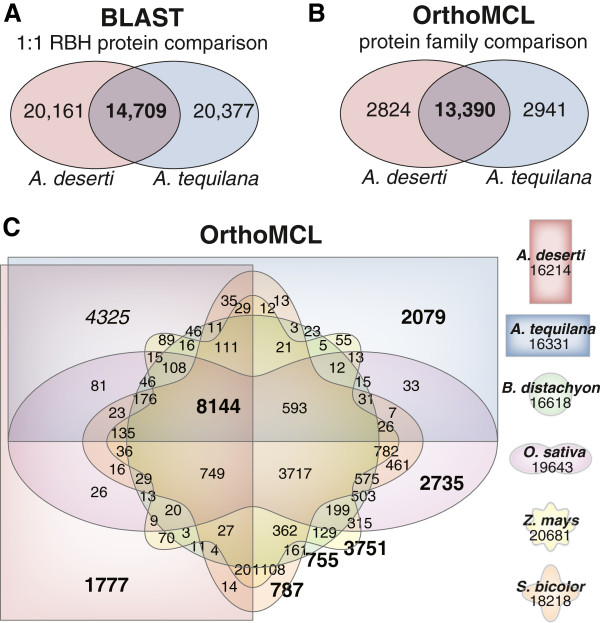
**Comparison of inferred proteomes of agaves and additional plant species. (A)** Diagram of BLASTP-based 1:1 reciprocal best hit (RBH) proteins shared between agaves*.***(B)** Diagram of OrthoMCL-defined protein families shared between agaves. **(C)** Diagram of OrthoMCL-defined plant orthologous-group protein families (Plant OGs) shared between agaves and 4 additional monocotyledonous plant species. Shape and color used for each species is noted with the total number of Plant OGs within each species.

We identified a core set of 14,709 reciprocal best hit (RBH) protein pairs between the *A. deserti* and *A. tequilana* using BLASTP (Figure 3A). The lengths of these RBH proteins correlate strongly (Pearson *r* = 0.90) and local alignments demonstrate a median amino acid sequence identity of 98.1%. This high correspondence between independently assembled datasets further supports assembly accuracy and suggests that a major proportion of the *A. deserti* and *A. tequilana* proteomes are shared between the two species. To further investigate proteomic similarity between agaves, we clustered the *Agave* proteomes into protein families using OrthoMCL [34]. Most (~80%) of the OrthoMCL-defined protein families in *A. deserti* and *A. tequilana* are common to both species (Figure 3B).

To further substantiate the *de novo Agave* transcriptomes and perform comparative analyses, *Agave* proteomes were also clustered by OrthoMCL with the proteomes of 11 additional plant kingdom species obtained from Phytozome [35] (hereafter, Phytozome Tester Set, or PTS, see Methods for details). The PTS includes both monocotyledonous and dicotyledonous plants and plants exhibiting C3 or C4 photosynthesis, but no other CAM plants as no high-quality datasets are currently available in Phytozome. Between *A. deserti*, *A. tequilana*, and the 11 species within the PTS, we obtained 48,133 unique plant protein orthologous groups (hereafter, Plant OGs) from a total of 381,050 proteins [36].

Using OrthoMCL data, we first compared protein lengths between the inferred proteomes of the PTS and our *de novo Agave* assemblies to address transcript contig completeness. There are 12,346 Plant OGs shared between either *A. deserti* or *A. tequilana* and at least one member of the PTS. These 12,346 Plant OGs encompass 55,676 *Agave* proteins and 173,611 proteins from the 11 species in the PTS (data available online [36]). The median lengths of *Agave* and PTS proteins within each of the 12,346 Plant OGs correlate highly (Pearson *r* = 0.85) and overall demonstrate 1:1 correspondence in protein lengths (best-fit slope = 0.9942) (Additional file 1: Figure S3A). The median length of *Agave* proteins within the set is ~11% shorter than that of the PTS (*Agave*, 356 amino acids; PTS, 389 amino acids; Student’s *t*-test *p*-value < 0.05) (Additional file 1: Figure S3B).

To estimate *Agave* proteome completeness, we compared the inferred *A. tequilana* and *A. deserti* proteomes to those of 4 monocotyledonous grass species in the PTS: *Brachypodium distachyon*, *Oryza sativa*, *Sorghum bicolor*, and *Zea mays*. An Edwards-Venn diagram of Plant OGs (Figure 3C) demonstrates that 8144 of 13,203 (61.7%) of protein families common to the 4 grass species are shared with agaves despite approximately 120 million years of evolution separating these grasses (order Poales [37]) and agaves (order Asparagales [37]) [38,39].

The 4325 Plant OGs common to both agaves but absent in four grass species (Figure 3C) represent either *Agave* protein families not present in grasses or protein families with enough sequence diversity to escape orthology detection by OrthoMCL. Gene ontology (GO) enrichment indicates regulatory diversity separates agaves from other monocots (Additional file 1: Table S7). Abundant transcription factor families within this set include MYB (InterPro IPR014778; 84 Plant OGs), ethylene response factor-domain (AP2/ERF-domain, IPR001471; 48 Plant OGs), C3HC4 Zinc finger (IPR018957; 44 Plant OGs), and WRKY (IPR003657; 41 Plant OGs). This agave-specific set also includes Hsp20-type heat shock proteins (IPR002068; 18 Plant OGs), suggestive of sequence divergence in these agave proteins regulating responses to heat.

### *Agave* protein families are of comparable size to those in other plant species

Agaves may have adapted to hot, arid environments through expansion of protein families involved in abiotic stress resistance. A comparison of 41,425 OrthoMCL-defined protein families common to any member of the PTS species and either *Agave* species failed to discover significantly smaller or larger orthologous protein families in agaves (Wilcoxon rank sum test Benjamini-Hochberg corrected *p*-values > 0.05). Furthermore, no significant expansion of obvious candidate protein families, such as heat shock proteins (HSPs) [40], heat-shock transcription factors (HSFs) [41], and dehydrins [42] was observed. Thus, using our clustering methodologies, we found no significant expansion of gene families within *Agave* species suggestive of adaptation to xeric environments. However, the lack of significantly underrepresented PlantOGs supports the completeness of our *de novo* transcriptome assemblies.

### Identifying polymorphisms in *A. deserti* and *A. tequilana*

Both wild-growing *A. deserti* and traditionally cultivated *A. tequilana* are expected to harbor significant amounts of heterozygosity. Furthermore, though both *A. tequilana* and *A. deserti* are cytological diploids [43], recent work indicates agaves are paleopolyploids resulting from two distinct tetraploidization events [27], potentially leading to the presence of highly similar paralagous loci in their genomes. While these issues can potentially complicate the *de novo* assembly of transcriptomes due to the consolidation of transcripts originating from distinct alleles or paralagous loci into single transcript contigs, these expected polymorphisms provide opportunities to demonstrate the utility of *de novo* transcriptomes to develop strategies for marker assisted breeding. We attempted to identify loci displaying evidence of combined assembly of polymorphic alleles and/or paralagous genes by mapping reads back to the reference consensus assembly and identifying single-nucleotide polymorphisms (SNPs) or insertions/deletions (indels).

Analysis identified 30,035 (33.9%) *A. deserti* loci and 66,701 (47.8%) *A. tequilana* loci as having 1 or more high-confidence polymorphism when compared to the reference Illumina *de novo* assembly (Additional file 1: Figure S4A). The median number of polymorphisms (SNPs or indels) per kilobase (hereafter, PPK) is significantly different between the two species, with 2.066 PPK in *A. deserti* and 4.39 PPK in *A. tequilana* (Wilcoxon rank sum test *p*-value < 0.05). Of loci exhibiting polymorphisms, 16,838 (56.1%) *A. deserti* and 34732 (52.1%) *A. tequilana* loci are protein-coding. In *A. deserti*, non-coding loci exhibit a higher median PPK than coding loci (2.9 vs. 1.6 PPK, respectively, Wilcoxon Rank Sum test *p-*value < 0.05) (Additional file 1: Figure S4), however this was not observed in *A. tequilana* (4.46 PPK coding, 4.29 PPK non-coding, Wilcoxon Rank Sum test *p*-value > 0.05) (Additional file 1: Figure S4B). Full datasets are available online [36].

### Mining *Agave* proteins for adaptations to xeric environments

In *ex vivo* experiments, *Agave* leaf cells can survive temperatures up to 64.7°C [23], suggesting molecular and cellular adaptations contribute to heat tolerance in a manner independent of *Agave* physiological and morphological adaptations. Though computational prediction of protein thermostability from primary structure alone is not completely accurate [44], we tested for protein adaptations to thermal stress using a streamlined version of Thermorank [44] (Additional file 1: Figure S5A, Methods). However, we found no signatures of global, proteome-wide thermotolerance adaptation in agaves (Additional file 1: Figure S5B). Independent tests of *Agave* proteins within OrthoMCL-defined PlantOGs failed to find agave proteins with significantly higher thermostability than others within Phytozome Tester Set (Wilcoxon rank sum test Benjamini-Hochberg adjusted *p*-values > 0.05).

### *Agave* transposable elements are transcriptionally active

Most plant transposable elements (TEs) are transcriptionally silent [45], they constitute a significant proportion of *Agave* genomes [28] and contribute to the creation of genetic diversity in many plants [46] including *Agave*[47,48]. We identified TE-like sequences in agaves (Methods) (Additional file 1: Table S8), the majority of which are derived from retrotransposons (Additional file 1: Table S8). Very few TE annotations encompass entire contigs (Additional file 1: Figure S6), with only 332 contigs in *A. tequilana* and 171 in *A. deserti* entirely covered by a TE annotation (±10 nt from each end). Nearly half of all TE annotations (46.6%) encompass only the 5′ or 3′ end of transcript contigs (Additional file 1: Figure S7), suggesting that transcription initiation or termination can occur within TEs.

### Transcriptome profiles of *Agave* tissues are distinguished by physiological function

To examine tissue-specific differences in transcriptome profiles, we analyzed the data sets used for *de novo* transcriptome assembly based on their tissue of origin (Additional file 1: Table S1). As expected, transcriptome profiles differ between *Agave* tissues in proportion to their respective physiological functions (Additional file 1: Tables S9 and S10, Figures S8 and S9). For example, very small transcriptome differences are observed between adjacent sections of the *A. deserti* leaf (*r* = 0.98), while the largest differences are observed between roots and leaves in *A. tequilana* (*r* = 0.42) (Additional file 1: Table S9) and between the distal tip of *A. deserti* leaves and roots (*r* = 0.39) (Additional file 1: Table S10).

In *A. deserti*, we observed consistent higher expression of 13,961 transcripts in samples derived from folded leaves and meristematic tissues (Methods) (Additional file 1: Figure S9). GO-terms enriched within these transcripts include functions related to DNA synthesis, lipid and membrane synthesis, and targeting of proteins to cellular membranes (Additional file 1: Table S11), all activities typically enriched in actively growing cells within developing leaves and meristems. Two enriched GO terms (DNA integration (GO:0015074) and RNA-dependent DNA replication (GO:0006278)) relate to TE biological functions, and 1524 of the 13,961 transcripts (11%) are TE-like sequences. Further analysis confirms TE-like sequences generally exhibit highest levels of expression in *A. deserti* folded leaf and meristem tissues (Additional file 1: Figure S10), consistent with developmental relaxation of transposable element silencing (DRTS), observed in meristematic tissues of other monocotyledonous plants [49].

### Transcriptomic insights into the *A. deserti* proximal-distal leaf axis

**Figure 4:**
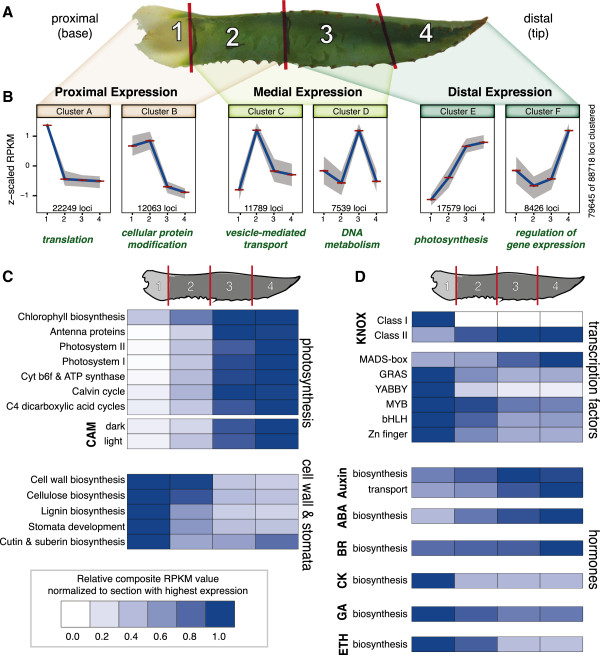
**Transcriptomic analysis of the *A. deserti *leaf proximal-distal axis. (A)** One of the *A. deserti* leaves used for analysis, indicating proximal-distal (PD) sections 1–4. **(B)** Six major *K*-means clusters of gene expression along the PD axis. Clusters are grouped by highest expression in proximal, medial, or distal tissues. Blue lines connect mean *z*-scaled RPKM values, shaded areas represent the 25th and 75th percentiles. Green text beneath each cluster denotes the most significantly enriched GO term in each cluster. **(C, D)** Heatmaps of composite gene expression for indicated biological processes. Data supporting figure are in Additional file 2. See text for details.

Monocotyledonous leaves develop along a proximal-distal (PD) gradient, maturing from the distal end (leaf tip) to the proximal end (base) [50], and therefore offer an opportunity to assess developmentally regulated gene expression [51]. As leaves are involved in bioenergy-relevant traits (e.g. photosynthesis) and directly face environmental stresses, we sought to gain general insights into the biology of *A. deserti* leaves (Figure 4A). *A. deserti* loci were divided into 6 major clusters based on expression patterns across four PD sections of the leaf (Figure 4A, 4B) (Methods). GO enrichment analyses identifies biological functions enriched in each cluster (Additional file 1: Tables S12–S17). Clusters A and B, including genes with highest expression at the leaf base, include many loci encoding regulatory proteins, as well as proteins involved in cell wall biogenesis, cellulose synthesis, and carbohydrate synthesis. Clusters E and F, containing genes expressed highest in distal portions of the leaf, include GO terms related to photosynthesis, chlorophyll biosynthesis, and additional regulatory proteins. Taken together, clustering data support the notion that growth and organizational processes occur in basal portions of the leaf while many important energy-related metabolic processes occur at the distal end. General patterns of gene expression for select biological functions were visualized along the leaf PD axis (Figure 4C and Additional file 2). Genes involved in photosynthesis are universally expressed higher in the distal portion of the leaf (Figure 4C), including genes involved in the diurnal shuttling of CO_2_ in CAM plants (Additional file 1: Table S18). This suggests that medial-distal portions of *A. deserti* juvenile leaves are the major site of photosynthesis. On the other hand, the basal portion of the leaf is the site of many developmental processes, including cell wall, lignin, and cellulose biosynthesis, stomata development and patterning, and epicuticular wax and suberin biosynthesis (Figure 4C).

We also examined expression of several classes of developmentally-important plant transcription factors and hormones (Figure 4D). Most transcription factor families are expressed highest at the leaf base. Notable exceptions to this pattern are the Class II KNOX genes, which tend to have broad patterns of expression [52], and MADS-box transcription factors, which regulate diverse developmental processes [53]. Hormone synthesis genes are also expressed in gradients along the leaf PD axis (Figure 4D) consistent with their roles in leaves [54,55]. We observed general PD patterns for auxin, abscisic acid (ABA), brassinosteroids (BR), cytokinin (CK), gibberellin (GA), and ethylene (ETH) hormone biosynthesis (Figure 4D). Taken together, the general patterns observed along the PD axis of the *A. deserti* leaf mirror those seen in the monocotyledonous grass *Zea mays*[51], with transcription factors regulating developmental processes expressed mostly at the leaf base, and functions of the mature leaf, such as photosynthesis, occur more toward the distal end.

## Discussion and conclusion

The transcriptomes of two agaves adapted to semi-arid (*A. tequilana*) and xeric (*A. deserti*) environments offer new resources in which to study CAM photosynthesis and other physiological adaptations to prolonged drought and heat. Comparisons of the *Agave de novo* transcriptome assemblies to other agave sequences and cross-species proteomic comparisons suggest the *de novo* assemblies are largely complete and accurate. However, the transcriptomes alone provide limited insight into how agaves survive in their environments. For example, though we have identified known genes central to CAM biochemistry in *Agave* (Additional file 1: Table S18), a full understanding of CAM biology in *Agave* requires studying the regulation of photosynthetic genes in response to physiological and environmental conditions [25,56,57]. This highlights the need to further functional understanding of *Agave* transcriptomes through experimentation. Our reference transcriptomes enable molecular investigations of agaves under environmentally controlled conditions to further elaborate the coordinated gene expression underlying CAM, drought resistance, and heat tolerance. As agaves are distinguished from other monocotyledonous plants by regulatory diversity (Additional file 1: Table S7), agave responses to stress may differ from other plants in novel ways.

A simple hypothesis is that agaves adapted to their environments by the expansion of gene families, and the *Agave* transcriptomes allow preliminary analyses of gene duplication. However, our analysis of inferred *Agave* proteomes and those of 11 other plant species in the PTS found no solid evidence of gene family expansion in agaves. We cannot, however, rule out the possibility of undetected gene family expansion for two reasons. Firstly, OrthoMCL, our clustering algorithm of choice, is relatively strict compared to alternative clustering algorithms [58], potentially leading to false negative results. Secondly, as agaves are paleopolyploids [27] and gene duplication events cannot be resolved cleanly without a reference genome, expansion of gene families with highly similar sequences will go undetected. More detailed studies of the extent and nature of *Agave* gene duplications will need to be addressed with a completed genome sequence.

Analyses of the inferred *Agave* proteomes by Thermorank also failed to find solid evidence of large-scale protein adaptations to thermal stress. In fact, *Agave* proteomes appear to be no more or less thermostable than those of other land plants (Additional file 1: Figure S5B). Interestingly, the proteome of the green algae *Chlamydomonas reinhardtii* showed the lowest overall thermostability of the 11 species within Phytozome Tester Set (Additional file 1: Figure S5B)—an expected result given its aquatic habitat—suggesting Thermorank can detect broad differences in protein thermostability. However, to detect more subtle differences between land plant proteomes, more robust methods using protein structure and molecular dynamic simulations [59] may be needed to resolve more subtle protein adaptations to thermal stress.

The *de novo* transcriptome assemblies are useful to develop molecular markers for further efforts in agave breeding or molecular studies, and we used our data to generate tables of polymorphic sites in the standardized VCF format [60]. Given the species of *Agave* studied here are primarily outcrossing [61,62] and more often reproduce clonally [5], we expected to find a large number of polymorphisms within the transcriptome. Consistent with this hypothesis, large percentages of both the *A. deserti* and *A. tequilana* loci display SNPs and indels. We also observed a significantly higher frequency of polymorphisms in *A. tequilana* than *A. deserti*, consistent with the source of the materials, as the *A. deserti* sequence data was generated from two sibling plants, while *A. tequilana* sequence data was generated from a population of individuals (Additional file 1: Table S1). The true number of heterozygous loci may be much higher as alleles not exhibiting equal expression may escape detection with our RNA-seq analyses.

Our analysis of gene expression along the proximal-distal axis of the juvenile *A. deserti* leaf demonstrates core classes of genes and biological processes are similar to those observed in *Zea mays*[51], supporting evolutionary conservation of monocotyledonous leaf development models [50]. A contrast of *A. deserti* to *Zea mays* is the expression pattern of MADS-box transcription factors. MADS-box genes can vary widely in expression and function in *Agave* floral structures and meristems [63], but their role in leaf developmental or metabolic processes remains unknown. The location of auxin biosynthesis occurs at a comparatively more distal portion of the blade in *A. deserti* than *Z. mays*[51]. These distinctions between *Agave* and *Zea mays* could be related to morphological differences between the two species: unlike maize leaves, *A. deserti* leaves are lanceolate-shaped with marginal spines [5] and have distinct parenchyma (water storing) and chlorenchyma (photosynthetic) tissues characteristic of succulent plants [19]. Such differences in key developmental transcription factors and hormones are perhaps not unexpected as these may be major determinants of *Agave* morphological adaptations to xeric environments.

Our *Agave* transcriptomes exemplify the power of *de novo* transcriptome assembly from short-read RNA-seq data [31], which provides both a high-quality sequence resource and insights through transcriptome profiling. Leveraging annotation tools and the scientific work from model plant species facilitated insights into the biology *Agave*. This rapid production of comprehensive sequence resources for additional species of industrial and biotechnological interest is needed to meet challenges of climate change and bioenergy development [64]. Our *de novo Agave* transcriptome assemblies provide a guide for such future *de novo* transcriptome assembly projects. Additional improvements in sequencing length, accuracy, cost, and throughput will make *de novo* transcriptome assembly an increasingly attractive option for rapid transcriptome exploration.

## Methods

### Plant materials

*A. tequilana* plants were collected from an *A. tequilana* plantation in Guanajuato, Mexico. Leaf, root, and stem tissue was collected from 2 different adult plants, each approximately 4 years of age. Juvenile plants from the same field, each approximately 1 year old, were dissected. Equal weights of juvenile roots, leaves, and stems were pooled prior to RNA preparation. *A. deserti* juveniles were obtained from a local commercial provider (Berkeley, CA) and verified using morphological keys [5]. Plants and tissues were dissected as described (Additional file 1: Table S1, Figure S1). *A. deserti* tissues were collected from well-watered plants near mid-day.

### Molecular methods

*Agave tequilana* RNA was extracted from tissues as described previously [65]. *A. deserti* RNA was prepared with modifications as follows. Tissues were finely sliced and immediately frozen in liquid nitrogen. Approximately 3 g of plant tissue were finely ground using liquid nitrogen in a mortar and pestle. 7.5 ml Trizol (Invitrogen, Carlsbad, CA) and 1.5 ml chloroform was added and tissue was homogenized. Homegenate was incubated for 10 min at room temperature and centrifuged 4000 g. Aqueous phase was removed, mixed with 1 volume of a 1:1 phenol:chloroform, and centrifuged at 4000 g. Resulting aqueous phase was mixed with an equal volume of isopropanol and 1/10 volume 5 M NaCl. RNA was precipitated at −20°C overnight, then centrifuged at 10,000 g. RNA pellet was suspended in 500 μl RNAse-free H_2_O. Phenol:Chloroform extraction was repeated as above. Aqueous phase was mixed with 0.6 volumes of 7 M LiCl and incubated at −20°C for 40 minutes prior to centrifugation at full speed in a table top microcentrifuge. RNA pellet was rinsed in 70% ethanol, air-dried briefly, and suspended in 250 μl H_2_O.

### Illumina short-read library construction and sequencing

Each library construction was initiated with 25 μg total RNA. Polyadenylated RNA was selected using the μMACS mRNA isolation kit (Miltenyi Biotec, Auburn, CA) and repeated as necessary until rRNA constituted less than 5% of the remaining purified mRNA before hydrolysis into 250 and 500 nt fragments using RNA Fragmentation Reagents (Ambion, Austin TX). First strand cDNA synthesis was performed using SuperScript II Reverse Transcriptase (Invitrogen, Carlsbad, CA) primed with 3 μg random hexamers and 2.5 mM dNTPs. 2nd strand cDNA was prepared in a 100 μl reaction with 2U RNAseH, 40U DNA Pol I, and 10U DNA ligase with 0.3 mM of each dNTP (with dUTP in place of dTTP). Samples were incubated for 2 hours at 16°C. Ten units T4 DNA polymerase was added and incubation was continued for an additional 5 minutes. 2nd strand cDNAs were size selected to either 250 or 500 bp using the Pippin Prep system (Sage Science, Beverly, MA). Indexed TruSeq libraries were constructed using manufacturer directions (Illumina, San Diego, CA). dUTP-labeled strands were destroyed using AmpErase uracil N-glycosylase (Applied Biosystems, Foster City, CA). Libraries were amplified through 10 cycles of PCR using Illumina guidelines. Sequencing was performed at the DOE Joint Genome Institute on an Illumina HiSeq 2000 with TruSeq SBS-v3 reagents (Illumina).

### Long-read sequencing

Libraries for Pacific Biosciences single molecule real time (SMRT) sequencing were prepared from *A. tequilana* 2nd strand cDNAs (see above). Library were constructed according to manufacturers’ guidelines (Pacific Biosciences, Menlo Park, CA) and sequenced on 5 SMRT cells for a total of 751,460 reads. Reads were filtered to remove library artifacts, resulting in 9913 read sequences composed of 27,787 subreads. Filtered Pacific Biosciences subread sequences ≥ 300 nt were corrected with 114,901,038 *A. tequilana* Illumina reads using methods described previously [66]. From this, 4767 successfully corrected, high quality PacBio subreads were returned. To compare the Pacific Biosciences sequencing to the Illumina de novo assembly, corrected PacBio subreads were aligned to the Illumina *A. tequilana* assembly using BLAT [33] with a minimum threshold of 90% sequence identity.

### *De novo* transcriptome assembly and analyses

*de novo* transcriptome assembly of Illumina sequence was performed by Rnnotator [29]. Transcript contigs were binned into loci based on a minimum of 200 bp sequence overlap as determined by an all-vs-all comparison using Vmatch [67]. Following assembly, transcripts were assigned an RPKM [68] value based on the number of uniquely mapping reads aligning to each transcript using BWA [69]. Each transcript version per locus was numbered according to its relative abundance for that locus (with version 1 being the most abundant). Transcripts present at less than 10% of the version 1 transcript were noted as potential precursor transcripts. Transcripts are named by their respective locus, version (isoform) number, raw RPKM and precursor flag; e.g. Locus1v2rpkm3.45_PRE is the 2nd most abundant isoform of Locus 1 with an RPKM of 3.45, marked as a precursor transcript. Additional details about the design and operation of Rnnotator can be found online [70].

### Filtering assemblies for high-confidence *Agave* transcriptomes

MEGAN v4.621 build 27 [71] was used to identify *de novo* assembled transcript contigs with homology to plant sequences and filtered RepeatMasker v. open-3.2.9 [72] and DeconSeq v 0.4.1 [73] with a contaminating match equivalent to > = 94% identity over 90% of the contig length. Sequences unidentified by MEGAN, Deconseq, or Repeatmasker were either retained or removed from the *Agave* datasets using their abundance (measured in RPKM) assuming most RNA in the sample originates from agave. Thresholds were defined by the lower quartile RPKM of high-confidence plant contigs. Contigs meeting or exceeding this RPKM were retained within the agave datasets (RPKM > = 0.42 for *A. deserti*, RPKM > = 1.2 for *A. tequilana*)*.*

### Protein prediction, annotation, and clustering

Open reading frames were annotated using EMBOSS getorf [74] with a maximum length of 1 × 10^6^ and a minimum length of 30 amino acids. Working-set proteomes include only proteins encoded on the + strand of v1 (most abundant) transcript isoforms, where each protein must be at least 76 aa in length with a CDS encompassing > = 50% of transcript length. Minimum protein lengths of 76 aa represents the 5th percentile of protein lengths within the Phytozome Tester Set (below). Pfam, Interpro, and GO annotation was performed using InterProScan [75]. KEGG annotation [76] was retrieved using KAAS [77]. TEs were identified using RepeatMasker (version open-3.2.9) [72] with RepBase Update 2009-06-04 [78].

### Phytozome tester set and protein clustering

The Phytozome Tester Set (PTS) includes select proteomes from Phytozome v8 [35]: *Arabidopsis thaliana* (TAIR release 10), *Brachypodium distachyon* (JGI v1.0 8X assembly of *Bd21* and MIPS/JGI v1.2 annotation), *Chlamydomonas reinhardtii* (Augustus u10.2 annotation of JGI assembly v4), Glycine max (JGI Glyma1.0 annotation of Glyma1 assembly), *Medicago truncatula* (Medicago Genome Sequence Consortium release Mt 3.0), *Oryza sativa* japonica (MSU Release 7.0), *Populus trichocarpa* (JGI release v2.0, annotation v2.2), *Ricinus communis* (TIGR release 0.1), *Setaria italica* (JGI 8.3X chromosome-scale assembly release 2.0, annotation version 2.1), *Sorghum bicolor* (Sbi1.4 models from MIPS/PASA on v1.0 assembly), and *Zea mays* (Maize Genome Project 5b.60 B73). Proteins were binned into orthologous groups (Plant OGs) by OrthoMCL v2.0.3 [34] using default settings.

### SNP and indel detection

All paired-end reads from *A. deserti* libraries (1,231,372,300 reads) and randomly selected *A. tequilana* reads (993,931,796 reads) were used to detect SNPs and indels (polymorphisms). *A. deserti* reads were aligned to the *A. deserti* v1 transcript contigs and *A. tequilana* reads were aligned to the *A. tequilana* v1 transcript contigs using BWA [69]. Polymorphisms within the v1 transcripts, serving as a proxy for a genomic locus, were called using SAMtools mpileup [60]. Based on the quality value distribution of SNPs and indels (Additional file 1: Figure S4A), only those with a quality score of 999 were considered for further analysis, minimizing low-confidence polymorphism calls from poor sequence quality or low-coverage.

### Protein thermostability prediction

In order to computationally predict thermostability for large protein datasets, we used the core scoring function of Thermorank [44] to assign a thermostability score to each protein as follows:

$$\begin{array}{l} \mathrm{Thermostability}=\left(K•0.75\right)+\left(E•0.2\right)+\left(\mathrm{Pos}•0.8\right)+\left(\mathrm{Chg}•0.2\right)\\ \;\;\;+\left(\mathrm{Sml}•-0.2\right)+\left(\mathrm{Tiny}•-0.2\right)+\left(A•-0.3\right)\\ \;\;\;+\left(Q•-0.1\right)+\left(T•-0.02\right)+\left(\mathrm{ASA}•0.9\right) \end{array}$$

Where *K* is the molar fraction of lysine, *E* is the molar fraction of glutamic acid, *Pos* is the molar fraction of positively charged amino acids (R, H and K), *Chg* is the molar fraction of charged amino acids (D, E, H, K, and R), *Sml* is the molar fraction of ‘small’ amino acids (A, C, D, G, N, P, S, T, and V), *Tiny* is the molar fraction of ‘tiny’ amino acids (A, C, G, S, and T), *T* is the molar fraction of threonine. *ASA* is calculated as follows: The residue surface accessible area for each amino acid residue (R) in a hypothetical Gly-R-Gly tripeptide was indexed to data obtained by Chothia [79]. The sum surface area for the peptide is divided by the number of amino acids to obtain an average residue surface area. The average surface area (possible minimum of 75 square angstroms (Å^2^) and maximum of 255 Å^2^[79]) is divided by 180 Å^2^ (the range between 75 Å^2^ and 255 Å^2^) to create a dimensionless value between 0 and 1. A test of 5000 artificial peptide sequences of random length and amino acid composition found high correlation between our thermostability score generator and Thermorank (Pearson *r* = 0.873, Additional file 1: Figure S5A).

### Protein family size and thermostability analyses

Detection of *Agave* OrthMCL-defined PlantOG family memberships, and Thermorank scores were determined by performing a Wilcoxon rank sum text against data obtained from the Phytozome Tester Set. Prior to analysis of PlantOG membership, protein identifiers from agaves and the Phytozome Tester Set were parsed to select non-redundant representative proteomes with a single version 1 (or similarly-labeled) representative protein model per locus. Final *p*-values were corrected for multiple comparisons by the Benjamini-Hochberg procedure [80].

### RNA-seq expression analysis and *K*-means clustering

Contigs containing rRNA-like sequences as determined by BLASTN [81] (E-value ≤ 10) against the SILVA v108 database [82] were removed from reference transcriptomes prior to expression analyses. Reads were trimmed to 36 nt and mapped to reference transcriptome using BWA [69]. The number of reads uniquely aligning to each transcript was normalized by the total number of uniquely-aligning reads in the sample, divided by the length of the uniquely mappable portion of each transcript to obtain an RPKM value [68]. Q-values were obtained as described [83]. *Z*-scaled locus RPKM values were grouped by *K*-means clustering, 6 clusters were chosen based on the ‘least within group sum of squares’ method [84]. All enrichment analysis was performed using BiNGO [85] with default settings (hypergeometric test with Benjamini-Hochberg *p*-value correction [80]).

### Data availability

Reads are available through the NCBI Sequence Read Archive (SRA), study accessions [GenBank:SRP019885] (*A. tequilana*) and [GenBank:SRP019506] (*A. deserti*). *Agave* transcriptome assembly contigs meeting NCBI requirements are deposited at the Transcriptome Shotgun Assembly (TSA) accessions *A. tequilana*: [GenBank:GAHU00000000]; *A. deserti*: [GenBank:GAHT00000000]. Full sequence assemblies, annotations, OrthoMCL clustering, and expression data for both agave datasets as described are available at the Dryad Digital Repository [36].

## Abbreviations

Bp: Base pair; BWA: Burrows-Wheeler Aligner; CAM: Crassulacean acid metabolism; DRTS: Developmental relaxation of transposable element silencing; Gbp: Gigabasepair(s); GO: Gene ontology; nt: nucleotide(s); RBH: Reciprocal best hit; RPKM: Reads per kilobase of exon model per million mapped reads; TE: Transposable element.

## Competing interests

S.G. has received travel reimbursement funds from Illumina Inc. (San Diego, CA). The authors declared that they have no competing interest.

## Authors’ contributions

SG, JM, ZW, and AV designed research. SG and JM performed research. JS and MA-J contributed biological materials. SG and AV wrote the manuscript. All authors read and approved the final manuscript.

## Supplementary Material

Additional file 1: Contains supplementary tables and figures referenced in the main text. Click here for file

Additional file 2: **Data supporting Figure** 4**.** Click here for file

## References

[B1] Borland AM Griffiths H Hartwell J Smith JAC Exploiting the potential of plants with crassulacean acid metabolism for bioenergy production on marginal lands J Exp Bot 2009 60 10 2879 2896 19395392 10.1093/jxb/erp118

[B2] Nobel PS Achievable productivities of vertain CAM plants - basis for high values compared with C3 and C4 plants New Phytol 1991 119 2 183 205 10.1111/j.1469-8137.1991.tb01022.x 33874131

[B3] Woodhouse RM Williams JG Nobel PS Simulation of plant temperature and water loss by the desert succulent, *Agave deserti* Oecologia 1983 57 3 291 297 10.1007/BF00377170 28309353

[B4] Nobel PS Water relations and photosynthesis of a desert CAM plant, *Agave deserti* Plant Physiol 1976 58 4 576 582 16659721 10.1104/pp.58.4.576 PMC543285

[B5] Gentry HS Agaves of continental North America 1982 Tucson, Ariz: University of Arizona Press

[B6] Gates DM Keegan HJ Schleter JC Weidner VR Spectral properties of plants Appl Optics 1965 4 1 11

[B7] Boom A Damste JSS de Leeuw JW Cutan, a common aliphatic biopolymer in cuticles of drought-adapted plants Org Geochem 2005 36 4 595 601

[B8] Wattendorff J Holloway PJ Studies on the ultrastructure and histochemistry of plant cuticles - the cuticular membrane of *Agave americana* L. *in situ* Ann Bot-London 1980 46 1 13

[B9] North GB Nobel PS Root-soil contact for the desert succulent *Agave deserti* in wet and drying soil New Phytol 1997 135 1 21 29 10.1046/j.1469-8137.1997.00620.x 33863144

[B10] North GB Brinton EK Garrett TY Contractile roots in succulent monocots: convergence, divergence and adaptation to limited rainfall Plant Cell Environ 2008 31 8 1179 1189 18507804 10.1111/j.1365-3040.2008.01832.x

[B11] Nobel PS Desert wisdom/agaves and cacti : CO2, water, climate change 2010 New York: iUniverse

[B12] Garcia-Moya E Romero-Manzanares A Nobel PS Highlights for *Agave* productivity Gcb Bioenergy 2011 3 1 4 14

[B13] Somerville C Youngs H Taylor C Davis SC Long SP Feedstocks for lignocellulosic biofuels Science 2010 329 5993 790 792 20705851 10.1126/science.1189268

[B14] Davis AS Dohleman F Long SP The global potential for *Agave* as a biofuel feedstock Gcb Bioenergy 2011 3 1 68 78

[B15] Cedeno M Tequila production Crit Rev Biotechnol 1995 15 1 1 11 7736598 10.3109/07388559509150529

[B16] Valenzuela-Zapata AG Nabhan GP Tequila : a natural and cultural history 2003 Tucson: University of Arizona Press

[B17] Distilled Spirits Council of the United States U.S. tequila market at a glance 2011 Washington, D.C: Distilled Spirits Council of the United States

[B18] Valenzuela A A new agenda for blue agave landraces: food, energy and tequila Gcb Bioenergy 2011 3 1 15 24

[B19] Nobel PS Environmental biology of agaves and cacti 1988 Cambridge; New York: Cambridge University Press

[B20] Nobel PS Hartsock TL Temperature, water, and PAR influences on predicted and measured productivity of *Agave deserti* at various elevations Oecologia 1986 68 2 181 185 10.1007/BF00384785 28310125

[B21] Zabriskie JG Plants of Deep Canyon and the Central Coachella Valley, California 1979 Riverside, CA: Philip L. Boyd Deep Canyon Desert Research Center, Univeristy of California, Riverside

[B22] Jordan PW Nobel PS Infrequent establishment of seedlings of *Agave deserti* (Agavaceae) in the northwestern Sonoran desert Am J Bot 1979 66 9 1079 1084

[B23] Nobel PS Smith SD High and low temperature tolerances and their relationships to distribution of agaves Plant Cell Environ 1983 6 9 711 719

[B24] Nobel PS Productivity of *Agave deserti* - measurement by dry-weight and monthly prediction using physiological-responses to environmental parameters Oecologia 1984 64 1 1 7 10.1007/BF00377535 28311630

[B25] Nobel PS Valenzuela AG Environmental responses and productivity of the CAM plant, *Agave tequilana* Agr Forest Meteorol 1987 39 4 319 334

[B26] Palomino G Dolezel J Mendez I Rubluo A Nuclear genome size analysis of *Agave tequilana Weber* Caryologia 2003 56 1 37 46

[B27] McKain MR Wickett N Zhang Y Ayyampalayam S McCombie WR Chase MW Pires JC Depamphilis CW Leebens-Mack J Phylogenomic analysis of transcriptome data elucidates co-occurrence of a paleopolyploid event and the origin of bimodal karyotypes in Agavoideae (Asparagaceae) Am J Bot 2012 99 2 397 406 22301890 10.3732/ajb.1100537

[B28] Bousios A Saldana-Oyarzabal I Valenzuela-Zapata AG Wood C Pearce SR Isolation and characterization of *Ty1-copia* retrotransposon sequences in the blue agave (*Agave tequilana Weber* var. *azul*) and their development as SSAP markers for phylogenetic analysis Plant Sci 2007 172 2 291 298

[B29] Martin J Bruno VM Fang Z Meng X Blow M Zhang T Sherlock G Snyder M Wang Z Rnnotator: an automated *de novo* transcriptome assembly pipeline from stranded RNA-seq reads BMC Genomics 2010 11 663 21106091 10.1186/1471-2164-11-663 PMC3152782

[B30] Zerbino DR Birney E Velvet: algorithms for de novo short read assembly using de Bruijn graphs Genome Res 2008 18 5 821 829 18349386 10.1101/gr.074492.107 PMC2336801

[B31] Martin JA Wang Z Next-generation transcriptome assembly Nat Rev Genet 2011 12 10 671 682 21897427 10.1038/nrg3068

[B32] Eid J Fehr A Gray J Luong K Lyle J Otto G Peluso P Rank D Baybayan P Bettman B Real-time DNA sequencing from single polymerase molecules Science 2009 323 5910 133 138 19023044 10.1126/science.1162986

[B33] Kent WJ BLAT–the BLAST-like alignment tool Genome Res 2002 12 4 656 664 11932250 10.1101/gr.229202 PMC187518

[B34] Fischer S Brunk BP Chen F Gao X Harb OS Iodice JB Shanmugam D Roos DS Stoeckert CJ Jr Using OrthoMCL to assign proteins to OrthoMCL-DB groups or to cluster proteomes into new ortholog groups Curr Protoc Bioinformatics 2011 Chapter 6 Unit 6 12 11 19 10.1002/0471250953.bi0612s35 PMC3196566 21901743

[B35] Goodstein DM Shu S Howson R Neupane R Hayes RD Fazo J Mitros T Dirks W Hellsten U Putnam N Phytozome: a comparative platform for green plant genomics Nucleic Acids Res 2012 40 Database issue D1178 D1186 22110026 10.1093/nar/gkr944 PMC3245001

[B36] Dryad Digital Repository[http://datadryad.org/resource/doi:10.5061/dryad.h5t68]

[B37] Angiosperm Phylogeny Group An update of the angiosperm phylogeny group classification for the orders and families of flowering plants: APG III Bot J Linn Soc 2009 161 105 121

[B38] Janssen T Bremer K The age of major monocot groups inferred from 800 + rbcL sequences Bot J Linn Soc 2004 146 4 385 398

[B39] Magallon S Castillo A Angiosperm diversification through time Am J Bot 2009 96 1 349 365 21628193 10.3732/ajb.0800060

[B40] Timperio AM Egidi MG Zolla L Proteomics applied on plant abiotic stresses: role of heat shock proteins (HSP) J Proteomics 2008 71 4 391 411 18718564 10.1016/j.jprot.2008.07.005

[B41] Scharf KD Berberich T Ebersberger I Nover L The plant heat stress transcription factor (Hsf) family: structure, function and evolution Biochim Biophys Acta 2012 1819 2 104 119 22033015 10.1016/j.bbagrm.2011.10.002

[B42] Hanin M Brini F Ebel C Toda Y Takeda S Masmoudi K Plant dehydrins and stress tolerance: versatile proteins for complex mechanisms Plant Signal Behav 2011 6 10 1503 1509 21897131 10.4161/psb.6.10.17088 PMC3256378

[B43] Granick EB A karyosystematic study of the genus *Agave* Am J Bot 1944 31 5 283 298

[B44] Li Y Middaugh CR Fang J A novel scoring function for discriminating hyperthermophilic and mesophilic proteins with application to predicting relative thermostability of protein mutants BMC Bioinforma 2010 11 62 10.1186/1471-2105-11-62 PMC3098108 20109199

[B45] Lisch D Bennetzen JL Transposable element origins of epigenetic gene regulation Curr Opin Plant Biol 2011 14 2 156 161 21444239 10.1016/j.pbi.2011.01.003

[B46] Lisch D How important are transposons for plant evolution? Nat Rev Genet 2012 14 1 49 61 23247435 10.1038/nrg3374

[B47] Torres-Moran MI Escoto-Delgadillo M Molina-Moret S Rivera-Rodriguez DM Velasco-Ramirez AP Infante D Portillo L Assessment of genetic fidelity among *Agave tequilana* plants propagated asexually via rhizomes versus in vitro culture Plant Cell Tiss Org 2010 103 3 403 409

[B48] Infante D Molina S Demey JR Gamez E Asexual genetic variability in Agavaceae determined with inverse sequence-tagged repeats and amplification fragment length polymorphism analysis Plant Mol Biol Rep 2006 24 2 205 217

[B49] Martinez G Slotkin RK Developmental relaxation of transposable element silencing in plants: functional or byproduct? Curr Opin Plant Biol 2012 15 496 502 23022393 10.1016/j.pbi.2012.09.001

[B50] Freeling M A conceptual framework for maize leaf development Dev Biol 1992 153 1 44 58 1516751 10.1016/0012-1606(92)90090-4

[B51] Li P Ponnala L Gandotra N Wang L Si Y Tausta SL Kebrom TH Provart N Patel R Myers CR The developmental dynamics of the maize leaf transcriptome Nat Genet 2010 42 12 1060 1067 21037569 10.1038/ng.703

[B52] Hake S Smith HM Holtan H Magnani E Mele G Ramirez J The role of knox genes in plant development Annu Rev Cell Dev Biol 2004 20 125 151 15473837 10.1146/annurev.cellbio.20.031803.093824

[B53] Smaczniak C Immink RG Angenent GC Kaufmann K Developmental and evolutionary diversity of plant MADS-domain factors: insights from recent studies Development 2012 139 17 3081 3098 22872082 10.1242/dev.074674

[B54] McSteen P Auxin and monocot development Cold Spring Harb Perspect Biol 2010 2 3 a001479 20300208 10.1101/cshperspect.a001479 PMC2829952

[B55] Blein T Hasson A Laufs P Leaf development: what it needs to be complex Curr Opin Plant Biol 2010 13 1 75 82 19853496 10.1016/j.pbi.2009.09.017

[B56] Silvera K Neubig KM Whitten WM Williams NH Winter K Cushman JC Evolution along the crassulacean acid metabolism continuum Funct Plant Biol 2010 37 11 995 1010

[B57] Hartsock TL Nobel PS Watering converts a CAM plant to daytime CO_2_ uptake Nature 1976 262 574 576

[B58] Chen F Mackey AJ Vermunt JK Roos DS Assessing performance of orthology detection strategies applied to eukaryotic genomes PLoS One 2007 2 4 e383 17440619 10.1371/journal.pone.0000383 PMC1849888

[B59] Sterpone F Melchionna S Thermophilic proteins: insight and perspective from in silico experiments Chem Soc Rev 2012 41 5 1665 1676 21975514 10.1039/c1cs15199a PMC3775309

[B60] Li H Handsaker B Wysoker A Fennell T Ruan J Homer N Marth G Abecasis G Durbin R Genome Project Data Processing S The sequence alignment/map format and SAMtools Bioinformatics 2009 25 16 2078 2079 19505943 10.1093/bioinformatics/btp352 PMC2723002

[B61] Molina-Freaner F Eguiarte LE The pollination biology of two paniculate agaves (Agavaceae) from northwestern Mexico: contrasting roles of bats as pollinators Am J Bot 2003 90 7 1016 1024 21659200 10.3732/ajb.90.7.1016

[B62] Escobar-Guzman RE Hernandez FZ Vega KG Simpson J Seed production and gametophyte formation in Agave tequilana and Agave americana Botany 2008 86 11 1343 1353

[B63] Delgado Sandoval Sdel C Abraham Juarez MJ Simpson J *Agave tequilana* MADS genes show novel expression patterns in meristems, developing bulbils and floral organs Sex Plant Reprod 2012 25 1 11 26 22012076 10.1007/s00497-011-0176-x

[B64] Rubin EM Genomics of cellulosic biofuels Nature 2008 454 7206 841 845 18704079 10.1038/nature07190

[B65] Martinez-Hernandez A Mena-Espino ME Herrera-Estrella AH Martinez-Hernandez P Construcción de bibliotecas de ADNc y análisis de expresión génica por RT-PCR en agaves Revista Latinoamericana de Química 2010 38 21 42

[B66] Koren S Schatz MC Walenz BP Martin J Howard JT Ganapathy G Wang Z Rasko DA McCombie WR Jarvis ED Hybrid error correction and de novo assembly of single-molecule sequencing reads Nat Biotechnol 2012 30 7 693 700 22750884 10.1038/nbt.2280 PMC3707490

[B67] Vmatch[http://www.vmatch.de]

[B68] Mortazavi A Williams BA McCue K Schaeffer L Wold B Mapping and quantifying mammalian transcriptomes by RNA-Seq Nat Methods 2008 5 7 621 628 18516045 10.1038/nmeth.1226 PMC13303166

[B69] Li H Durbin R Fast and accurate short read alignment with Burrows-Wheeler transform Bioinformatics 2009 25 14 1754 1760 19451168 10.1093/bioinformatics/btp324 PMC2705234

[B70] Rnnotator on google code[https://sites.google.com/a/lbl.gov/rnnotator/]

[B71] Huson DH Mitra S Ruscheweyh HJ Weber N Schuster SC Integrative analysis of environmental sequences using MEGAN4 Genome Res 2011 21 9 1552 1560 21690186 10.1101/gr.120618.111 PMC3166839

[B72] Smit AFA Hubley R Green P Repeatmasker open-3.0 http://www.repeatmasker.org. 1996–2010

[B73] Schmieder R Edwards R Fast identification and removal of sequence contamination from genomic and metagenomic datasets PLoS One 2011 6 3 e17288 21408061 10.1371/journal.pone.0017288 PMC3052304

[B74] Rice P Longden I Bleasby A EMBOSS: the European molecular biology open software suite Trends Genet 2000 16 6 276 277 10827456 10.1016/s0168-9525(00)02024-2

[B75] Zdobnov EM Apweiler R InterProScan–an integration platform for the signature-recognition methods in InterPro Bioinformatics 2001 17 9 847 848 11590104 10.1093/bioinformatics/17.9.847

[B76] Kanehisa M Goto S Sato Y Furumichi M Tanabe M KEGG for integration and interpretation of large-scale molecular data sets Nucleic Acids Res 2012 40 Database issue D109 D114 22080510 10.1093/nar/gkr988 PMC3245020

[B77] Moriya Y Itoh M Okuda S Yoshizawa AC Kanehisa M KAAS: an automatic genome annotation and pathway reconstruction server Nucleic Acids Res 2007 35 Web Server issue W182 W185 17526522 10.1093/nar/gkm321 PMC1933193

[B78] Jurka J Kapitonov VV Pavlicek A Klonowski P Kohany O Walichiewicz J Repbase update, a database of eukaryotic repetitive elements Cytogenet Genome Res 2005 110 1–4 462 467 16093699 10.1159/000084979

[B79] Chothia C Nature of accessible and buried surfaces in proteins J Mol Biol 1976 105 1 1 14 994183 10.1016/0022-2836(76)90191-1

[B80] Benjamini Y Hochberg Y Controlling the false discovery rate: a practical and powerful aproach to multiple testing J R Stat Soc 1995 57 1 289 300

[B81] Altschul SF Gish W Miller W Myers EW Lipman DJ Basic local alignment search tool J Mol Biol 1990 215 3 403 410 2231712 10.1016/S0022-2836(05)80360-2

[B82] Pruesse E Quast C Knittel K Fuchs BM Ludwig W Peplies J Glockner FO SILVA: a comprehensive online resource for quality checked and aligned ribosomal RNA sequence data compatible with ARB Nucleic Acids Res 2007 35 21 7188 7196 17947321 10.1093/nar/gkm864 PMC2175337

[B83] Herbert JM Stekel D Sanderson S Heath VL Bicknell R A novel method of differential gene expression analysis using multiple cDNA libraries applied to the identification of tumour endothelial genes BMC Genomics 2008 9 153 18394197 10.1186/1471-2164-9-153 PMC2346479

[B84] Everitt BS Hothorn T A handbook of statistical analyses using R 2006 1 Boca Raton, FL: Chapman and Hall

[B85] Maere S Heymans K Kuiper M BiNGO: a Cytoscape plugin to assess overrepresentation of gene ontology categories in biological networks Bioinformatics 2005 21 16 3448 3449 15972284 10.1093/bioinformatics/bti551

