# Supplementary material for: De novo transcriptome assembly of drought tolerant CAM plants, Agave deserti and Agave tequilana
Source: BMC Genomics. 2013 Aug 19;14:563. doi: 10.1186/1471-2164-14-563 (PMC3765226; doi:10.1186/1471-2164-14-563)
Supplement: Additional file 1: — Contains supplementary tables and figures referenced in the main text. [file 1471-2164-14-563-S1.docx]

**Supplementary Information for Gross *et al.***

Supplementary Tables and Figures

**Supplemental Tables:**

Table S1: Description of sampled *A. tequilana* and *A. deserti* tissues

Table S2: Illumina sequencing summary for *A. tequilana*

Table S3: Illumina sequencing summary for *A. deserti*

Table S4: Summary of assemblies prior to removal of contaminating transcripts

Table S5: Summary of *Agave* transcriptomes following removal of contaminating transcripts

Table S6: Final summary of assembled non-agave contaminating sequences

Table S7: Enriched GO terms unique to *Agave* and common annotations in *A. deserti* and *A. tequilana*

Table S8: Summary of transposable elements and repetitive sequences

Table S9: Transcripts differentially expressed between *A. tequilana* samples

Table S10: Transcripts differentially expressed between *A. deserti* samples

Table S11: Enriched GO terms in transcripts consistently highly expressed in developing leaves and meristems

Table S12: Gene ontology terms enriched in *A. deserti* leaf cluster A

Table S13: Gene ontology terms enriched in *A. deserti* leaf cluster B

Table S14: Gene ontology terms enriched in *A. deserti* leaf cluster C

Table S15: Gene ontology terms enriched in *A. deserti* leaf cluster D

Table S16: Gene ontology terms enriched in *A. deserti* leaf cluster E

Table S17: Gene ontology terms enriched in *A. des*erti leaf cluster F

Table S18: High confidence proteins composing core pathways of CAM photosynthesis

**Supplemental Figures:**

Figure S1: *Agave* *deserti* plants used for transcriptome assembly and expression analysis

Figure S2: Histograms of *A. tequilana* PacBio subread lengths.

Figure S3: Agave protein lengths compared to the Phytozome Tester Set

Figure S4: Detection of polymorphisms in the *Agave* transcriptomes

Figure S5: Analysis of plant proteomes for adaptations to thermal stress

Figure S6: Violin plots of *Agave* transcript coverage by RepeatMasker

Figure S7: Position of transposable element annotations within transcript contigs

Figure S8: Differential expression of transcripts between *A. tequilana* tissues

Figure S9: Differential expression of transcripts between *A. deserti* tissues

Figure S10: Expression of transposable elements in agaves

**Table S1: Description of sampled *A. tequilana* and *A. deserti* tissues**

| **Species** | **Tissue** | **Description** | **No. of pooled individuals** | **Specific individuals sampled** |
| --- | --- | --- | --- | --- |
| *A. tequilana* | Roots | Roots from a fully mature adult plant | n.r. | n.a. |
| *A. tequilana* | Leaf | Medial section of a mature leaf from an adult plant | n.r. | n.a. |
| *A. tequilana* | Stem | Apical portion of stem below folded leaves. Adult plant. | n.r. | n.a. |
| *A. tequilana* | Juveniles | Equal weights of leaves, stem tissue, and roots from several individuals pooled together. | n.r. | n.a. |
| *A. deserti* | Leaf Section 1 | Proximal end of 2 fully developed juvenile leaves. Non-photosynthetic. See text for details. | 1 | 2 |
| *A. deserti* | Leaf Section 2 | Medial section of 2 leaf blades. See text for details. | 1 | 2 |
| *A. deserti* | Leaf Section 3 | Medial section of 2 leaf blades. See text for details. | 1 | 2 |
| *A. deserti* | Leaf Section 4 | Distal end of 2 fully developed juvenile leaves. See text for details. | 1 | 2 |
| *A. deserti* | Proximal Leaf | Basal half of fully developed juvenile leaf. Roughly equivalent to a combination of Leaf Sections 1 and 2 (above). | 1 | 2 |
| *A. deserti* | Ramets | 3 underground ramets derived from same mother plant. All ramet tissues used. | 1 | 1 |
| *A. deserti* | Folded leaves & meristem | Folded leaves and meristem tissue at center of rosette | 1 | 2 |
| *A. deserti* | Roots | Bulked roots | 2 | 1, 2 |

n.r.—not recorded. n.a.—not applicable

**Table S2: *A. tequilana* Illumina sequencing data summary**

| **Lane** | **Insert size** | **Sample** | **Q20**  **read 1** | **Q20**  **read 2** | **No. of reads** | **Percent adapter** | **Percent rRNA** | **Gigabasepairs** |
| --- | --- | --- | --- | --- | --- | --- | --- | --- |
| 1 | 500 bp | roots | 144 | 140 | 110,509,808 | 0.2 | 10.1 | 16.6 |
| 1 | 500 bp | stem | 143 | 136 | 68,157,118 | 0.1 | 4.5 | 10.2 |
| 1 | 500 bp | juveniles | 143 | 138 | 73,907,970 | 0.5 | 10.6 | 11.1 |
| 1 | 500 bp | leaf | 141 | 132 | 61,624,666 | 0.7 | 4.5 | 9.2 |
| 2 | 500 bp | roots | 144 | 138 | 90,422,342 | 0.2 | 9.9 | 13.6 |
| 2 | 500 bp | stem | 143 | 135 | 55,241,120 | 0.1 | 4.4 | 8.3 |
| 2 | 500 bp | juveniles | 143 | 137 | 59,470,722 | 0.6 | 10.2 | 8.9 |
| 2 | 500 bp | leaf | 142 | 131 | 50,322,742 | 0.6 | 4.3 | 7.5 |
| 3 | 250 bp | stem | 146 | 141 | 32,425,860 | 0.3 | 38.5 | 4.9 |
| 3 | 250 bp | root | 146 | 142 | 31,937,910 | 1.5 | 29.7 | 4.8 |
| 3 | 250 bp | leaf | 147 | 144 | 29,608,546 | 1.1 | 31.8 | 4.4 |
| 3 | 250 bp | root | 147 | 145 | 27,212,980 | 0.3 | 11.2 | 4.1 |
| 3 | 250 bp | leaf | 147 | 143 | 27,217,774 | 0.2 | 35.4 | 4.1 |
| 3 | 250 bp | juveniles | 145 | 140 | 30,474,368 | 1.7 | 38.1 | 4.6 |
| 3 | 250 bp | juveniles | 145 | 141 | 37,310,808 | 0.7 | 40.8 | 5.6 |
| 3 | 250 bp | stem | 148 | 145 | 30,714,130 | 0.3 | 4.8 | 4.6 |
| 4 | 250 bp | stem | 143 | 139 | 39,116,138 | 0.3 | 38.8 | 5.9 |
| 4 | 250 bp | root | 142 | 140 | 38,483,382 | 1.5 | 30.0 | 5.8 |
| 4 | 250 bp | leaf | 144 | 141 | 35,601,472 | 1.3 | 32.7 | 5.3 |
| 4 | 250 bp | root | 144 | 143 | 32,924,898 | 0.4 | 11.1 | 4.9 |
| 4 | 250 bp | leaf | 144 | 141 | 32,950,110 | 0.2 | 36.7 | 4.9 |
| 4 | 250 bp | juveniles | 142 | 140 | 36,662,480 | 1.5 | 37.6 | 5.5 |
| 4 | 250 bp | juveniles | 143 | 140 | 44,923,212 | 0.6 | 41.0 | 6.7 |
| 4 | 250 bp | stem | 145 | 143 | 37,042,316 | 0.2 | 4.7 | 5.6 |
| 5 | 250 bp | stem | 135 | 137 | 32,910,578 | 0.3 | 37.1 | 4.9 |
| 5 | 250 bp | root | 135 | 139 | 32,443,344 | 1.2 | 29.6 | 4.9 |
| 5 | 250 bp | leaf | 137 | 140 | 30,191,014 | 1.4 | 31.7 | 4.5 |
| 5 | 250 bp | root | 137 | 141 | 27,624,678 | 0.4 | 11.7 | 4.1 |
| 5 | 250 bp | leaf | 137 | 140 | 27,600,316 | 0.1 | 35.1 | 4.1 |
| 5 | 250 bp | juveniles | 134 | 137 | 30,984,620 | 1.6 | 37.2 | 4.6 |
| 5 | 250 bp | juveniles | 135 | 138 | 37,795,888 | 0.6 | 39.9 | 5.7 |
| 5 | 250 bp | stem | 137 | 141 | 31,339,726 | 0.2 | 4.5 | 4.7 |
| 6 | 250 bp | stem | 133 | 139 | 38,287,964 | 0.3 | 38.1 | 5.7 |
| 6 | 250 bp | root | 132 | 140 | 38,293,872 | 1.4 | 29.1 | 5.7 |
| 6 | 250 bp | leaf | 135 | 141 | 34,853,428 | 1.4 | 31.1 | 5.2 |
| 6 | 250 bp | root | 135 | 141 | 32,373,582 | 0.5 | 11.9 | 4.9 |
| 6 | 250 bp | leaf | 135 | 140 | 32,785,326 | 0.1 | 35.0 | 4.9 |
| 6 | 250 bp | juveniles | 133 | 139 | 36,079,160 | 1.4 | 36.5 | 5.4 |
| 6 | 250 bp | juveniles | 133 | 138 | 44,122,112 | 0.7 | 39.8 | 6.6 |
| 6 | 250 bp | stem | 135 | 142 | 36,989,528 | 0.2 | 4.9 | 5.5 |
| 7 | 250 bp | stem | 138 | 140 | 39,134,436 | 0.3 | 37.8 | 5.9 |
| 7 | 250 bp | root | 139 | 142 | 38,487,308 | 1.4 | 29.1 | 5.8 |
| 7 | 250 bp | leaf | 140 | 143 | 35,693,028 | 1.3 | 30.9 | 5.4 |
| 7 | 250 bp | root | 140 | 144 | 32,882,418 | 0.4 | 11.7 | 4.9 |
| 7 | 250 bp | leaf | 140 | 143 | 33,031,414 | 0.2 | 35.2 | 5.0 |
| 7 | 250 bp | juveniles | 137 | 141 | 36,707,046 | 1.7 | 37.3 | 5.5 |
| 7 | 250 bp | juveniles | 139 | 141 | 44,862,900 | 0.6 | 39.7 | 6.7 |
| 7 | 250 bp | stem | 141 | 144 | 37,093,208 | 0.2 | 4.6 | 5.6 |
| **Totals** |  |  |  |  | **1,956,829,766** |  |  | **293.5** |

Q20 read 1—average length at which 1^st^ Illumina read is still > Phred (Q) score of 20

Q20 read 2—average length at which 2^nd^ Illumina read is still > Phred (Q) score of 20

**Table S3: *A. deserti* Illumina sequencing data summary**

| **Lane** | **Insert size** | **Sample** | **Q20 read 1** | **Q20 read 2** | **No. of reads** | **Percent adapter** | **Percent rRNA** | **Gbp** |
| --- | --- | --- | --- | --- | --- | --- | --- | --- |
| 1 | 250 bp | Roots | 127 | 130 | 19,083,324 | 5.9% | 11.38% | 2.9 |
| 1 | 250 bp | Folded leaves & meristem | 132 | 138 | 22,795,730 | 2.1% | 13.70% | 3.4 |
| 1 | 250 bp | Leaf (Section 1) | 129 | 133 | 21,557,058 | 1.6% | 30.09% | 3.2 |
| 1 | 250 bp | Leaf (Section 2) | 130 | 134 | 23,349,962 | 1.0% | 27.71% | 3.5 |
| 1 | 250 bp | Leaf (Section 3) | 131 | 136 | 24,299,186 | 2.3% | 26.90% | 3.6 |
| 1 | 250 bp | Leaf (Section 4) | 129 | 133 | 22,569,596 | 3.4% | 18.28% | 3.4 |
| 1 | 250 bp | Ramets | 130 | 136 | 23,732,074 | 1.7% | 18.23% | 3.6 |
| 1 | 250 bp | Proximal leaf | 126 | 130 | 11,639,610 | 9.7% | 26.64% | 1.7 |
| 2 | 250 bp | Roots | 123 | 126 | 19,288,030 | 5.9% | 11.32% | 2.9 |
| 2 | 250 bp | Folded leaves & meristem | 128 | 130 | 23,024,774 | 2.2% | 13.70% | 3.5 |
| 2 | 250 bp | Leaf (Section 1) | 125 | 128 | 21,764,640 | 1.6% | 30.08% | 3.3 |
| 2 | 250 bp | Leaf (Section 2) | 126 | 129 | 23,646,002 | 0.9% | 27.70% | 3.5 |
| 2 | 250 bp | Leaf (Section 3) | 127 | 130 | 24,562,376 | 2.2% | 26.97% | 3.7 |
| 2 | 250 bp | Leaf (Section 4) | 125 | 128 | 22,797,490 | 3.5% | 18.33% | 3.4 |
| 2 | 250 bp | Ramets | 126 | 130 | 24,001,134 | 1.7% | 18.10% | 3.6 |
| 2 | 250 bp | Proximal leaf | 122 | 124 | 11,708,220 | 10.0% | 26.65% | 1.8 |
| 3 | 250 bp | Roots | 121 | 121 | 19,036,220 | 6.1% | 11.35% | 2.9 |
| 3 | 250 bp | Folded leaves & meristem | 126 | 122 | 22,723,684 | 2.2% | 13.63% | 3.4 |
| 3 | 250 bp | Leaf (Section 1) | 123 | 121 | 21,439,174 | 1.5% | 30.14% | 3.2 |
| 3 | 250 bp | Leaf (Section 2) | 124 | 121 | 23,289,156 | 1.0% | 27.62% | 3.5 |
| 3 | 250 bp | Leaf (Section 3) | 125 | 122 | 24,285,168 | 2.2% | 26.99% | 3.6 |
| 3 | 250 bp | Leaf (Section 4) | 123 | 121 | 22,496,844 | 3.4% | 18.26% | 3.4 |
| 3 | 250 bp | Ramets | 124 | 122 | 23,692,960 | 1.4% | 18.15% | 3.6 |
| 3 | 250 bp | Proximal leaf | 120 | 119 | 11,562,980 | 10.1% | 26.74% | 1.7 |
| 4 | 250 bp | Roots | 116 | 126 | 19,258,936 | 6.0% | 11.30% | 2.9 |
| 4 | 250 bp | Folded leaves & meristem | 120 | 130 | 22,950,486 | 2.1% | 13.70% | 3.4 |
| 4 | 250 bp | Leaf (Section 1) | 118 | 127 | 21,644,408 | 1.7% | 29.92% | 3.2 |
| 4 | 250 bp | Leaf (Section 2) | 118 | 128 | 23,504,408 | 1.0% | 27.53% | 3.5 |
| 4 | 250 bp | Leaf (Section 3) | 120 | 129 | 24,492,106 | 2.2% | 26.84% | 3.7 |
| 4 | 250 bp | Leaf (Section 4) | 118 | 128 | 22,668,626 | 3.5% | 18.36% | 3.4 |
| 4 | 250 bp | Ramets | 119 | 129 | 23,894,930 | 1.7% | 18.13% | 3.6 |
| 4 | 250 bp | Proximal leaf | 115 | 124 | 11,712,124 | 9.9% | 26.50% | 1.8 |
| 5 | 500 bp | Roots | 128 | 132 | 23,596,416 | 23.8% | 7.19% | 3.5 |
| 5 | 500 bp | Folded leaves & meristem | 140 | 147 | 21,995,340 | 4.9% | 9.66% | 3.3 |
| 5 | 500 bp | Leaf (Section 1) | 141 | 147 | 27,793,922 | 2.6% | 26.19% | 4.2 |
| 5 | 500 bp | Leaf (Section 2) | 137 | 143 | 21,876,468 | 2.4% | 19.77% | 3.3 |
| 5 | 500 bp | Leaf (Section 3) | 140 | 146 | 25,971,440 | 2.7% | 20.85% | 3.9 |
| 5 | 500 bp | Leaf (Section 4) | 134 | 138 | 20,849,818 | 14.3% | 13.05% | 3.1 |
| 5 | 500 bp | Ramets | 139 | 145 | 25,906,270 | 4.3% | 14.06% | 3.9 |
| 5 | 500 bp | Proximal leaf | 131 | 136 | 17,176,152 | 14.1% | 20.59% | 2.6 |
| 6 | 500 bp | Roots | 126 | 128 | 23,487,940 | 24.6% | 7.12% | 3.5 |
| 6 | 500 bp | Folded leaves & meristem | 132 | 146 | 21,703,898 | 5.1% | 9.63% | 3.3 |
| 6 | 500 bp | Leaf (Section 1) | 134 | 146 | 27,402,108 | 2.6% | 26.18% | 4.1 |
| 6 | 500 bp | Leaf (Section 2) | 130 | 142 | 21,524,500 | 2.5% | 19.78% | 3.2 |
| 6 | 500 bp | Leaf (Section 3) | 133 | 145 | 25,554,454 | 2.7% | 20.76% | 3.8 |
| 6 | 500 bp | Leaf (Section 4) | 127 | 136 | 20,625,582 | 14.6% | 12.92% | 3.1 |
| 6 | 500 bp | Ramets | 132 | 144 | 25,536,196 | 4.3% | 13.99% | 3.8 |
| 6 | 500 bp | Proximal leaf | 126 | 134 | 17,050,630 | 14.3% | 20.57% | 2.6 |
| 7 | 500 bp | Roots | 128 | 128 | 23,754,008 | 24.6% | 7.11% | 3.6 |
| 7 | 500 bp | Folded leaves & meristem | 139 | 146 | 21,907,656 | 5.1% | 9.58% | 3.3 |
| 7 | 500 bp | Leaf (Section 1) | 140 | 146 | 27,733,564 | 2.7% | 26.22% | 4.2 |
| 7 | 500 bp | Leaf (Section 2) | 137 | 142 | 21,721,284 | 2.3% | 19.72% | 3.3 |
| 7 | 500 bp | Leaf (Section 3) | 140 | 144 | 25,795,216 | 2.7% | 20.84% | 3.9 |
| 7 | 500 bp | Leaf (Section 4) | 133 | 136 | 20,895,284 | 14.5% | 12.99% | 3.1 |
| 7 | 500 bp | Ramets | 138 | 144 | 25,753,996 | 4.2% | 13.97% | 3.9 |
| 7 | 500 bp | Proximal leaf | 130 | 133 | 17,288,742 | 14.1% | 20.41% | 2.6 |
| **Totals** |  |  |  |  | **1,231,372,300** |  |  | **184.7** |

Q20 read 1—average length at which 1^st^ Illumina read is still > Phred (Q) score of 20

Q20 read 2—average length at which 2^nd^ Illumina read is still > Phred (Q) score of 20

**Table S4: Summary of assemblies prior to removal of contaminating transcripts**

| **Species** | **No. of loci** | **No. of transcripts** | **Avgerage transcripts / locus** | **N50 transcript length** | **Median Length** | **Mean Length** | **Min length** | **Max Length** | **Sum length of all transcripts** |
| --- | --- | --- | --- | --- | --- | --- | --- | --- | --- |
| *A. tequilana* | 227,941 | 306,836 | 1.3 | 1168 bp | 553 bp | 813.8 bp | 100 bp | 20,000 bp | 249,717,332 bp |
| *A. deserti* | 165,698 | 216,897 | 1.3 | 1118 bp | 498 bp | 730.3 bp | 100 bp | 21,906 bp | 158,405,421 bp |

**Table S5: Summary of *Agave* transcriptomes following removal of contaminating transcripts**

| **Species** | **No. of loci** | **No. of transcripts** | **Avgerage transcripts / locus** | **N50 transcript length** | **Median Length** | **Mean Length** | **Min length** | **Max Length** | **Sum length of all transcripts** |
| --- | --- | --- | --- | --- | --- | --- | --- | --- | --- |
| *A. tequilana* | 139,525 | 204,530 | 1.47 | 1387 bp | 739 bp | 1001.6 bp | 100 bp | 20,000 bp | 204.854,948 bp |
| *A. deserti* | 88,718 | 128,869 | 1.45 | 1323 bp | 758 bp | 970.2 bp | 100 bp | 21,906 bp | 125,032,917 bp |

**Table S6: Final summary of assembled non-agave contaminating sequences**

| **Species** | **No. of loci** | **No. of transcripts** | **Avgerage transcripts / locus** | **N50 transcript length** | **Median Length** | **Mean Length** | **Min length** | **Max Length** | **Sum length of all transcripts** |
| --- | --- | --- | --- | --- | --- | --- | --- | --- | --- |
| *A. tequilana* | 88,416 | 102,306 | 1.157098263 | 510 bp | 380 bp | 438.5 bp | 100 bp | 16,860 bp | 44,862,384 bp |
| *A. deserti* | 76,980 | 88,028 | 1.143517797 | 480 bp | 272 bp | 379.1 bp | 100 bp | 17,906 bp | 33,372,504 bp |

**Table S7: Enriched GO terms in protein families unique to *Agave*, with common annotations in *A. deserti* and *A. tequilana***

| **GO term** | **description** | ***Agave deserti*** | | | |  | ***Agave tequilana*** | | | |
| --- | --- | --- | --- | --- | --- | --- | --- | --- | --- | --- |
|  |  | **p-value** | **corrected p-value** | **cluster frequency** | **total frequency** |  | **p-value** | **corrected p-value** | **cluster frequency** | **total frequency** |
| GO:0009889 | regulation of biosynthetic process | 1.04E-50 | 2.28E-48 | 310/1442 21.4% | 1384/14979 9.2% |  | 1.17E-51 | 2.58E-49 | 321/1510 21.2% | 1479/16108 9.1% |
| GO:0031326 | regulation of cellular biosynthetic process | 1.04E-50 | 2.28E-48 | 310/1442 21.4% | 1384/14979 9.2% |  | 1.17E-51 | 2.58E-49 | 321/1510 21.2% | 1479/16108 9.1% |
| GO:0010556 | regulation of macromolecule biosynthetic process | 1.04E-50 | 2.28E-48 | 310/1442 21.4% | 1384/14979 9.2% |  | 1.17E-51 | 2.58E-49 | 321/1510 21.2% | 1479/16108 9.1% |
| GO:0010468 | regulation of gene expression | 2.38E-50 | 3.93E-48 | 310/1442 21.4% | 1389/14979 9.2% |  | 4.89E-51 | 6.49E-49 | 321/1510 21.2% | 1488/16108 9.2% |
| GO:0080090 | regulation of primary metabolic process | 2.66E-49 | 3.51E-47 | 319/1442 22.1% | 1467/14979 9.7% |  | 1.96E-51 | 3.26E-49 | 330/1510 21.8% | 1547/16108 9.6% |
| GO:0060255 | regulation of macromolecule metabolic process | 3.30E-49 | 3.63E-47 | 310/1442 21.4% | 1405/14979 9.3% |  | 2.36E-50 | 2.61E-48 | 321/1510 21.2% | 1498/16108 9.2% |
| GO:0031323 | regulation of cellular metabolic process | 7.81E-48 | 7.35E-46 | 322/1442 22.3% | 1510/14979 10.0% |  | 9.22E-50 | 8.73E-48 | 333/1510 22.0% | 1594/16108 9.8% |
| GO:0019222 | regulation of metabolic process | 1.62E-46 | 1.33E-44 | 322/1442 22.3% | 1530/14979 10.2% |  | 1.34E-48 | 1.11E-46 | 333/1510 22.0% | 1612/16108 10.0% |
| GO:0051252 | regulation of RNA metabolic process | 8.38E-45 | 5.52E-43 | 290/1442 20.1% | 1328/14979 8.8% |  | 5.94E-47 | 3.94E-45 | 304/1510 20.1% | 1426/16108 8.8% |
| GO:0006355 | regulation of transcription, DNA-dependent | 8.38E-45 | 5.52E-43 | 290/1442 20.1% | 1328/14979 8.8% |  | 5.94E-47 | 3.94E-45 | 304/1510 20.1% | 1426/16108 8.8% |
| GO:0045449 | regulation of transcription | 9.82E-45 | 5.88E-43 | 290/1442 20.1% | 1329/14979 8.8% |  | 6.94E-47 | 4.18E-45 | 304/1510 20.1% | 1427/16108 8.8% |
| GO:0051171 | regulation of nitrogen compound metabolic process | 3.00E-43 | 1.52E-41 | 299/1442 20.7% | 1415/14979 9.4% |  | 1.67E-46 | 8.53E-45 | 313/1510 20.7% | 1498/16108 9.2% |
| GO:0019219 | regulation of nucleobase, nucleoside, nucleotide and nucleic acid metabolic process | 3.00E-43 | 1.52E-41 | 299/1442 20.7% | 1415/14979 9.4% |  | 1.67E-46 | 8.53E-45 | 313/1510 20.7% | 1498/16108 9.2% |
| GO:0050794 | regulation of cellular process | 4.54E-39 | 2.14E-37 | 362/1442 25.1% | 1960/14979 13.0% |  | 2.33E-39 | 1.10E-37 | 375/1510 24.8% | 2102/16108 13.0% |
| GO:0050789 | regulation of biological process | 7.56E-38 | 3.32E-36 | 363/1442 25.1% | 1992/14979 13.2% |  | 3.31E-38 | 1.46E-36 | 376/1510 24.9% | 2134/16108 13.2% |
| GO:0065007 | biological regulation | 3.91E-35 | 1.61E-33 | 369/1442 25.5% | 2096/14979 13.9% |  | 3.14E-35 | 1.30E-33 | 382/1510 25.2% | 2248/16108 13.9% |
| GO:0032269 | negative regulation of cellular protein metabolic process | 1.11E-08 | 3.32E-07 | 15/1442 1.0% | 29/14979 0.1% |  | 3.55E-07 | 1.05E-05 | 14/1510 0.9% | 32/16108 0.1% |
| GO:0051248 | negative regulation of protein metabolic process | 1.11E-08 | 3.32E-07 | 15/1442 1.0% | 29/14979 0.1% |  | 3.55E-07 | 1.05E-05 | 14/1510 0.9% | 32/16108 0.1% |
| GO:0017148 | negative regulation of translation | 1.11E-08 | 3.32E-07 | 15/1442 1.0% | 29/14979 0.1% |  | 3.55E-07 | 1.05E-05 | 14/1510 0.9% | 32/16108 0.1% |
| GO:0006417 | regulation of translation | 1.98E-09 | 6.88E-08 | 20/1442 1.3% | 46/14979 0.3% |  | 3.80E-07 | 1.05E-05 | 17/1510 1.1% | 46/16108 0.2% |
| GO:0032268 | regulation of cellular protein metabolic process | 1.98E-09 | 6.88E-08 | 20/1442 1.3% | 46/14979 0.3% |  | 3.80E-07 | 1.05E-05 | 17/1510 1.1% | 46/16108 0.2% |
| GO:0010608 | posttranscriptional regulation of gene expression | 1.98E-09 | 6.88E-08 | 20/1442 1.3% | 46/14979 0.3% |  | 5.44E-07 | 1.44E-05 | 17/1510 1.1% | 47/16108 0.2% |
| GO:0051246 | regulation of protein metabolic process | 2.53E-08 | 7.25E-07 | 20/1442 1.3% | 52/14979 0.3% |  | 1.07E-06 | 2.74E-05 | 17/1510 1.1% | 49/16108 0.3% |
| GO:0009890 | negative regulation of biosynthetic process | 6.12E-08 | 1.55E-06 | 15/1442 1.0% | 32/14979 0.2% |  | 1.33E-06 | 3.05E-05 | 14/1510 0.9% | 35/16108 0.2% |
| GO:0031327 | negative regulation of cellular biosynthetic process | 6.12E-08 | 1.55E-06 | 15/1442 1.0% | 32/14979 0.2% |  | 1.33E-06 | 3.05E-05 | 14/1510 0.9% | 35/16108 0.2% |
| GO:0010558 | negative regulation of macromolecule biosynthetic process | 6.12E-08 | 1.55E-06 | 15/1442 1.0% | 32/14979 0.2% |  | 1.33E-06 | 3.05E-05 | 14/1510 0.9% | 35/16108 0.2% |
| GO:0010605 | negative regulation of macromolecule metabolic process | 1.67E-07 | 4.07E-06 | 15/1442 1.0% | 34/14979 0.2% |  | 1.99E-06 | 4.40E-05 | 14/1510 0.9% | 36/16108 0.2% |
| GO:0048523 | negative regulation of cellular process | 1.99E-07 | 4.67E-06 | 18/1442 1.2% | 48/14979 0.3% |  | 5.51E-06 | 0.000111 | 16/1510 1.0% | 49/16108 0.3% |
| GO:0031324 | negative regulation of cellular metabolic process | 2.97E-06 | 6.53E-05 | 15/1442 1.0% | 41/14979 0.2% |  | 4.24E-06 | 8.78E-05 | 14/1510 0.9% | 38/16108 0.2% |
| GO:0048519 | negative regulation of biological process | 4.07E-07 | 9.24E-06 | 18/1442 1.2% | 50/14979 0.3% |  | 7.40E-06 | 0.00014 | 16/1510 1.0% | 50/16108 0.3% |
| GO:0009892 | negative regulation of metabolic process | 5.90E-06 | 0.000125 | 15/1442 1.0% | 43/14979 0.2% |  | 6.05E-06 | 0.000118 | 14/1510 0.9% | 39/16108 0.2% |
| GO:0009607 | response to biotic stimulus | 1.25E-05 | 0.000257 | 11/1442 0.7% | 26/14979 0.1% |  | 3.74E-06 | 7.99E-05 | 11/1510 0.7% | 24/16108 0.1% |
| GO:0006633 | fatty acid biosynthetic process | 0.000396 | 0.00792 | 26/1442 1.8% | 134/14979 0.8% |  | 0.000199 | 0.00321 | 27/1510 1.7% | 139/16108 0.8% |
| GO:0006952 | defense response | 0.000658 | 0.0116 | 11/1442 0.7% | 38/14979 0.2% |  | 9.54E-05 | 0.00158 | 11/1510 0.7% | 32/16108 0.1% |
| GO:0006631 | fatty acid metabolic process | 0.00177 | 0.0278 | 28/1442 1.9% | 163/14979 1.0% |  | 0.000305 | 0.00482 | 30/1510 1.9% | 165/16108 1.0% |
| GO:0042545 | cell wall modification | 0.00116 | 0.0191 | 14/1442 0.9% | 59/14979 0.3% |  | 0.00206 | 0.031 | 17/1510 1.1% | 85/16108 0.5% |

**Table S8: Summary of transposable elements and repetitive sequences detected by RepeatMasker**

| **Repeat Class** | ***A. tequilana*** | | | | | | | |  | ***A. deserti*** | | | | | | | |
| --- | --- | --- | --- | --- | --- | --- | --- | --- | --- | --- | --- | --- | --- | --- | --- | --- | --- |
|  | **No. transcripts** | **No. loci** | **Transcript RPKM** | | | | | |  | **No. transcripts** | **No. loci** | **Transcript RPKM** | | | | | |
|  |  |  | **Min** | **1st Quartile** | **Median** | **Mean** | **3rd Quartile** | **Max** |  |  |  | **Min** | **1st Quartile** | **Median** | **Mean** | **3rd Quartile** | **Max** |
| DNA/En-Spm | 238 | 180 | 0 | 0.6 | 1 | 8.184 | 4.775 | 110.5 |  | 130 | 96 | 0 | 0.4125 | 1.36 | 16.75 | 11.08 | 310.6 |
| DNA/Harbinger | 21 | 17 | 0 | 0.3 | 0.6 | 3.262 | 3 | 37.7 |  | 13 | 11 | 0 | 0.21 | 0.54 | 5.069 | 1.01 | 42.91 |
| DNA/Helitron | 200 | 147 | 0 | 1.1 | 5.25 | 20.11 | 23.65 | 218.4 |  | 153 | 107 | 0 | 1.29 | 6.36 | 21.99 | 18.47 | 289.1 |
| DNA/MuDR | 217 | 148 | 0 | 0.8 | 2 | 10.76 | 6.4 | 258.8 |  | 159 | 109 | 0 | 0.405 | 1.08 | 9.025 | 5.07 | 166.8 |
| DNA/hAT-Ac | 308 | 227 | 0 | 0.6 | 0.9 | 3.146 | 1.5 | 122.8 |  | 117 | 82 | 0 | 0.43 | 0.7 | 4.781 | 1.62 | 171.4 |
| DNA/hAT-Tag1 | 219 | 143 | 0 | 0.4 | 0.6 | 1.084 | 1.1 | 15.9 |  | 72 | 47 | 0 | 0.2275 | 0.415 | 1.025 | 0.7275 | 7.69 |
| DNA/hAT-Tip100 | 127 | 97 | 0 | 0.4 | 0.7 | 1.037 | 1.1 | 17.1 |  | 60 | 53 | 0 | 0.2275 | 0.34 | 1.205 | 0.63 | 31.52 |
| LINE/L1 | 292 | 241 | 0 | 0.4 | 0.6 | 2.296 | 1 | 207.7 |  | 156 | 129 | 0 | 0.23 | 0.43 | 1.805 | 0.845 | 58.28 |
| LINE/RTE-BovB | 1603 | 1356 | 0 | 0.5 | 0.7 | 1.474 | 1 | 154.7 |  | 550 | 490 | 0 | 0.28 | 0.43 | 1.016 | 0.63 | 50.9 |
| LTR | 5 | 5 | 0.6 | 0.7 | 0.7 | 1.66 | 1 | 5.3 |  | 5 | 4 | 0 | 0.11 | 0.24 | 0.434 | 0.39 | 1.43 |
| LTR/Copia | 3988 | 3287 | 0 | 0.4 | 0.6 | 1.679 | 0.8 | 1763 |  | 1490 | 1150 | 0 | 0.19 | 0.32 | 2.278 | 0.53 | 1173 |
| LTR/Gypsy | 3572 | 2843 | 0 | 0.3 | 0.5 | 3.439 | 0.8 | 797.1 |  | 1468 | 1083 | 0 | 0.19 | 0.335 | 7.389 | 0.74 | 1509 |
| Low_complexity | 16322 | 13407 | 0 | 0.7 | 1.1 | 6.972 | 2.7 | 2110 |  | 6680 | 5474 | 0 | 0.55 | 1.24 | 11.48 | 6.52 | 916.9 |
| Satellite | 8 | 6 | 0 | 0.975 | 3.35 | 3.988 | 5.125 | 12.8 |  | 5 | 4 | 1.08 | 1.56 | 3.7 | 5.066 | 6.09 | 12.9 |
| Simple_repeat | 32153 | 25077 | 0 | 0.8 | 1.7 | 11.15 | 7.8 | 2715 |  | 21631 | 17368 | 0 | 0.78 | 2.69 | 14.52 | 10.6 | 6285 |

Detection of transposable elements detected using RepeatMasker v. open-3.2.9 using the *Zea mays* repeat models present in Repbase Update v 17.08.

**Table S9: Transcripts differentially expressed between *A. tequilana* samples**

| **Sample 1** | **Sample 2** | ***r*** | **Transcripts with *q*-value < 0.001** | |  | **Transcripts differentially expressed at 2X level** | | | | |  | **Transcripts differentially expressed at 10X level** | | | | |
| --- | --- | --- | --- | --- | --- | --- | --- | --- | --- | --- | --- | --- | --- | --- | --- | --- |
|  |  |  |  |  |  | **More abundant in Sample 1** | |  | **More abundant in Sample 2** | |  | **More abundant in Sample 1** | |  | **More abundant in Sample 2** | |
|  |  |  | **number** | **percent** |  | **number** | **percent** |  | **number** | **percent** |  | **number** | **percent** |  | **number** | **percent** |
| leaf | juvenile | 0.59 | 81083 | 39.8% |  | 18872 | 9.3% |  | 37099 | 18.2% |  | 13686 | 6.7% |  | 3117 | 1.5% |
| roots | juvenile | 0.87 | 77961 | 38.2% |  | 22077 | 10.8% |  | 22813 | 11.2% |  | 6168 | 3.0% |  | 4675 | 2.3% |
| roots | leaf | 0.42 | 97361 | 47.7% |  | 45677 | 22.4% |  | 27341 | 13.4% |  | 8311 | 4.1% |  | 19049 | 9.3% |
| stem | juvenile | 0.74 | 76276 | 37.4% |  | 14758 | 7.2% |  | 33115 | 16.2% |  | 12891 | 6.3% |  | 2462 | 1.2% |
| stem | leaf | 0.47 | 87103 | 42.7% |  | 34254 | 16.8% |  | 30222 | 14.8% |  | 10605 | 5.2% |  | 10414 | 5.1% |
| stem | roots | 0.60 | 94048 | 46.1% |  | 24973 | 12.2% |  | 40071 | 19.6% |  | 15650 | 7.7% |  | 5324 | 2.6% |

**Table S10: Transcripts differentially expressed between *A. deserti* samples**

| **Sample 1** | **Sample 2** | ***r*** | **Transcripts with q-value < 0.001** | |  | **Transcripts differentially expressed at 2X level** | | | | |  | **Transcripts differentially expressed at 10X level** | | | | |
| --- | --- | --- | --- | --- | --- | --- | --- | --- | --- | --- | --- | --- | --- | --- | --- | --- |
|  |  |  | **number** | **percent** |  | **More abundant in Sample 1** | |  | **More abundant in Sample 2** | |  | **More abundant in Sample 1** | |  | **More abundant in Sample 2** | |
|  |  |  |  |  |  | **number** | **percent** |  | **number** | **percent** |  | **number** | **percent** |  | **number** | **percent** |
| leaf_pt1 | leaf_pt2 | 0.82 | 34377 | 26.7% |  | 10215 | 7.9% |  | 9683 | 7.5% |  | 1862 | 1.4% |  | 2345 | 1.8% |
| leaf_pt1 | leaf_pt3 | 0.47 | 42302 | 32.9% |  | 16309 | 12.7% |  | 13392 | 10.4% |  | 3962 | 3.1% |  | 5175 | 4.0% |
| leaf_pt1 | leaf_pt4 | 0.44 | 42563 | 33.1% |  | 17058 | 13.3% |  | 13812 | 10.7% |  | 4302 | 3.3% |  | 5564 | 4.3% |
| leaf_pt1 | proximal leaf | 0.70 | 33462 | 26.0% |  | 10592 | 8.2% |  | 11351 | 8.8% |  | 2337 | 1.8% |  | 3495 | 2.7% |
| leaf_pt1 | ramets | 0.60 | 58008 | 45.1% |  | 21176 | 16.5% |  | 23539 | 18.3% |  | 10892 | 8.5% |  | 8080 | 6.3% |
| leaf_pt1 | roots | 0.65 | 55876 | 43.5% |  | 22796 | 17.7% |  | 20591 | 16.0% |  | 10794 | 8.4% |  | 9424 | 7.3% |
| leaf_pt1 | folded leaves | 0.55 | 60284 | 46.9% |  | 12935 | 10.1% |  | 33620 | 26.2% |  | 12532 | 9.7% |  | 5260 | 4.1% |
| leaf_pt2 | leaf_pt3 | 0.74 | 29612 | 23.0% |  | 9457 | 7.4% |  | 6969 | 5.4% |  | 509 | 0.4% |  | 1471 | 1.1% |
| leaf_pt2 | leaf_pt4 | 0.71 | 31335 | 24.4% |  | 10553 | 8.2% |  | 8117 | 6.3% |  | 884 | 0.7% |  | 1933 | 1.5% |
| leaf_pt2 | proximal leaf | 0.74 | 29436 | 22.9% |  | 9318 | 7.2% |  | 9455 | 7.4% |  | 2367 | 1.8% |  | 2726 | 2.1% |
| leaf_pt2 | ramets | 0.64 | 56461 | 43.9% |  | 20395 | 15.9% |  | 22953 | 17.9% |  | 11805 | 9.2% |  | 7245 | 5.6% |
| leaf_pt2 | roots | 0.64 | 55363 | 43.1% |  | 22506 | 17.5% |  | 20744 | 16.1% |  | 11580 | 9.0% |  | 9033 | 7.0% |
| leaf_pt2 | folded leaves | 0.55 | 59318 | 46.1% |  | 13875 | 10.8% |  | 32363 | 25.2% |  | 15790 | 12.3% |  | 4818 | 3.7% |
| leaf_pt3 | leaf_pt4 | 0.98 | 9560 | 7.4% |  | 1039 | 0.8% |  | 903 | 0.7% |  | 188 | 0.1% |  | 82 | 0.1% |
| leaf_pt3 | proximal leaf | 0.68 | 29163 | 22.7% |  | 7847 | 6.1% |  | 9794 | 7.6% |  | 3171 | 2.5% |  | 1146 | 0.9% |
| leaf_pt3 | ramets | 0.39 | 61040 | 47.5% |  | 22236 | 17.3% |  | 26897 | 20.9% |  | 14078 | 11.0% |  | 8578 | 6.7% |
| leaf_pt3 | roots | 0.39 | 59085 | 46.0% |  | 23673 | 18.4% |  | 24325 | 18.9% |  | 13636 | 10.6% |  | 10399 | 8.1% |
| leaf_pt3 | folded leaves | 0.44 | 60651 | 47.2% |  | 13592 | 10.6% |  | 33787 | 26.3% |  | 16033 | 12.5% |  | 3679 | 2.9% |
| leaf_pt4 | proximal leaf | 0.64 | 30270 | 23.5% |  | 8402 | 6.5% |  | 10797 | 8.4% |  | 3398 | 2.6% |  | 1293 | 1.0% |
| leaf_pt4 | ramets | 0.36 | 59405 | 46.2% |  | 21466 | 16.7% |  | 27002 | 21.0% |  | 13888 | 10.8% |  | 8891 | 6.9% |
| leaf_pt4 | roots | 0.36 | 57997 | 45.1% |  | 22792 | 17.7% |  | 24729 | 19.2% |  | 13506 | 10.5% |  | 10471 | 8.1% |
| leaf_pt4 | folded leaves | 0.40 | 59682 | 46.4% |  | 13699 | 10.7% |  | 33903 | 26.4% |  | 15774 | 12.3% |  | 3798 | 3.0% |
| proximal leaf | ramets | 0.55 | 52650 | 41.0% |  | 20354 | 15.8% |  | 21793 | 17.0% |  | 10658 | 8.3% |  | 7076 | 5.5% |
| proximal leaf | roots | 0.50 | 53421 | 41.6% |  | 23143 | 18.0% |  | 20632 | 16.0% |  | 11484 | 8.9% |  | 9980 | 7.8% |
| proximal leaf | folded leaves | 0.77 | 40866 | 31.8% |  | 7313 | 5.7% |  | 21738 | 16.9% |  | 9323 | 7.3% |  | 1699 | 1.3% |
| ramets | roots | 0.70 | 34236 | 26.6% |  | 10435 | 8.1% |  | 9247 | 7.2% |  | 1801 | 1.4% |  | 2664 | 2.1% |
| ramets | folded leaves | 0.51 | 77559 | 60.3% |  | 23086 | 18.0% |  | 41567 | 32.3% |  | 22897 | 17.8% |  | 11774 | 9.2% |
| roots | folded leaves | 0.39 | 78036 | 60.7% |  | 22259 | 17.3% |  | 44527 | 34.6% |  | 26026 | 20.2% |  | 13037 | 10.1% |

**Table S11: Enriched GO terms in transcripts consistently highly expressed in folded developing leaves and meristems**

| **goterm** | **description** | **p-value** | **Corrected p-value** | **Cluster frequency** | **Total frequency** |
| --- | --- | --- | --- | --- | --- |
| GO:0006259 | DNA metabolic process | 3.67E-125 | 0.00E+00 | 274/1257 21.7% | 895/21159 4.2% |
| GO:0015074 | DNA integration | 1.09E-86 | 2.93E-84 | 133/1257 10.5% | 283/21159 1.3% |
| GO:0006278 | RNA-dependent DNA replication | 4.03E-85 | 7.20E-83 | 130/1257 10.3% | 275/21159 1.2% |
| GO:0090304 | nucleic acid metabolic process | 2.98E-71 | 3.99E-69 | 311/1257 24.7% | 1815/21159 8.5% |
| GO:0006260 | DNA replication | 5.96E-70 | 6.39E-68 | 136/1257 10.8% | 381/21159 1.8% |
| GO:0006139 | nucleobase containing compound metabolic process | 1.72E-52 | 1.53E-50 | 320/1257 25.4% | 2278/21159 10.7% |
| GO:0034641 | cellular nitrogen compound metabolic process | 3.01E-38 | 2.31E-36 | 333/1257 26.4% | 2811/21159 13.2% |
| GO:0006807 | nitrogen compound metabolic process | 1.42E-37 | 9.49E-36 | 340/1257 27.0% | 2920/21159 13.8% |
| GO:0044260 | cellular macromolecule metabolic process | 3.76E-23 | 2.24E-21 | 558/1257 44.3% | 6667/21159 31.5% |
| GO:0043170 | macromolecule metabolic process | 5.36E-21 | 2.87E-19 | 606/1257 48.2% | 7558/21159 35.7% |
| GO:0007018 | microtubule-based movement | 6.08E-15 | 2.96E-13 | 33/1257 2.6% | 112/21159 0.5% |
| GO:0044238 | primary metabolic process | 1.04E-13 | 4.66E-12 | 739/1257 58.7% | 10310/21159 48.7% |
| GO:0007017 | microtubule-based process | 2.39E-13 | 9.84E-12 | 36/1257 2.8% | 148/21159 0.6% |
| GO:0044237 | cellular metabolic process | 1.33E-06 | 5.09E-05 | 623/1257 49.5% | 9127/21159 43.1% |
| GO:0034645 | cellular macromolecule biosynthetic process | 8.41E-06 | 3.00E-04 | 191/1257 15.1% | 2387/21159 11.2% |
| GO:0009059 | macromolecule biosynthetic process | 1.12E-05 | 3.74E-04 | 191/1257 15.1% | 2398/21159 11.3% |
| GO:0009405 | pathogenesis | 2.09E-04 | 6.60E-03 | 3/1257 0.2% | 3/21159 0.0% |
| GO:0006952 | defense response | 6.72E-04 | 1.86E-02 | 9/1257 0.7% | 42/21159 0.1% |
| GO:0045017 | glycerolipid biosynthetic process | 6.93E-04 | 1.86E-02 | 12/1257 0.9% | 69/21159 0.3% |
| GO:0046474 | glycerophospholipid biosynthetic process | 6.93E-04 | 1.86E-02 | 12/1257 0.9% | 69/21159 0.3% |
| GO:0009987 | cellular process | 8.38E-04 | 2.14E-02 | 752/1257 59.8% | 11749/21159 55.5% |
| GO:0006468 | protein amino acid phosphorylation | 1.04E-03 | 2.53E-02 | 168/1257 13.3% | 2254/21159 10.6% |
| GO:0006464 | protein modification process | 1.26E-03 | 2.94E-02 | 201/1257 15.9% | 2771/21159 13.0% |
| GO:0006506 | GPI anchor biosynthetic process | 1.44E-03 | 3.09E-02 | 11/1257 0.8% | 65/21159 0.3% |
| GO:0046489 | phosphoinositide biosynthetic process | 1.44E-03 | 3.09E-02 | 11/1257 0.8% | 65/21159 0.3% |
| GO:0006650 | glycerophospholipid metabolic process | 2.11E-03 | 4.20E-02 | 14/1257 1.1% | 99/21159 0.4% |
| GO:0046486 | glycerolipid metabolic process | 2.11E-03 | 4.20E-02 | 14/1257 1.1% | 99/21159 0.4% |
| GO:0008152 | metabolic process | 2.52E-03 | 4.83E-02 | 940/1257 74.7% | 15088/21159 71.3% |
| GO:0006505 | GPI anchor metabolic process | 2.66E-03 | 4.92E-02 | 11/1257 0.8% | 70/21159 0.3% |

**Table S12: Gene Ontology Terms Enriched in *A. deserti* Leaf Cluster A**

| **goterm** | **name** | **p-value** | **corrected_p-value** | **Cluster Frequency** | **Total Frequency** |
| --- | --- | --- | --- | --- | --- |
| GO:0006412 | translation | 2.35E-28 | 2.06E-25 | 417/3656 (11.4%) | 1068/14979 (7.1%) |
| GO:0034645 | cellular macromolecule biosynthetic process | 1.63E-20 | 7.15E-18 | 606/3656 (16.5%) | 1814/14979 (12.1%) |
| GO:0009059 | macromolecule biosynthetic process | 3.08E-20 | 9.02E-18 | 607/3656 (16.6%) | 1822/14979 (12.1%) |
| GO:0010467 | gene expression | 1.17E-15 | 2.56E-13 | 508/3656 (13.8%) | 1545/14979 (10.3%) |
| GO:0044249 | cellular biosynthetic process | 7.33E-13 | 1.29E-10 | 765/3656 (20.9%) | 2549/14979 (17.0%) |
| GO:0009058 | biosynthetic process | 1.66E-10 | 2.42E-08 | 824/3656 (22.5%) | 2836/14979 (18.9%) |
| GO:0010468 | regulation of gene expression | 7.33E-10 | 9.19E-08 | 434/3656 (11.8%) | 1389/14979 (9.2%) |
| GO:0060255 | regulation of macromolecule metabolic process | 1.82E-09 | 1.84E-07 | 436/3656 (11.9%) | 1405/14979 (9.3%) |
| GO:0006355 | regulation of transcription, DNA-dependent | 2.63E-09 | 1.84E-07 | 414/3656 (11.3%) | 1328/14979 (8.8%) |
| GO:0051252 | regulation of RNA metabolic process | 2.63E-09 | 1.84E-07 | 414/3656 (11.3%) | 1328/14979 (8.8%) |
| GO:0045449 | regulation of transcription, DNA-dependent | 2.93E-09 | 1.84E-07 | 414/3656 (11.3%) | 1329/14979 (8.8%) |
| GO:0010556 | regulation of macromolecule biosynthetic process | 2.94E-09 | 1.84E-07 | 429/3656 (11.7%) | 1384/14979 (9.2%) |
| GO:0009889 | regulation of biosynthetic process | 2.94E-09 | 1.84E-07 | 429/3656 (11.7%) | 1384/14979 (9.2%) |
| GO:0031326 | regulation of cellular biosynthetic process | 2.94E-09 | 1.84E-07 | 429/3656 (11.7%) | 1384/14979 (9.2%) |
| GO:0044267 | cellular protein metabolic process | 3.45E-08 | 2.01E-06 | 967/3656 (26.4%) | 3465/14979 (23.1%) |
| GO:0044260 | cellular macromolecule metabolic process | 6.81E-08 | 3.73E-06 | 1276/3656 (34.9%) | 4696/14979 (31.3%) |
| GO:0080090 | regulation of primary metabolic process | 2.23E-07 | 1.15E-05 | 439/3656 (12.0%) | 1467/14979 (9.7%) |
| GO:0051171 | regulation of nitrogen compound metabolic process | 3.14E-07 | 1.45E-05 | 424/3656 (11.5%) | 1415/14979 (9.4%) |
| GO:0019219 | regulation of nucleobase-containing compound metabolic process | 3.14E-07 | 1.45E-05 | 424/3656 (11.5%) | 1415/14979 (9.4%) |
| GO:0019222 | regulation of metabolic process | 1.34E-06 | 5.87E-05 | 450/3656 (12.3%) | 1530/14979 (10.2%) |
| GO:0031323 | regulation of cellular metabolic process | 2.20E-06 | 9.20E-05 | 443/3656 (12.1%) | 1510/14979 (10.0%) |
| GO:0043170 | macromolecule metabolic process | 5.50E-06 | 2.19E-04 | 1412/3656 (38.6%) | 5328/14979 (35.5%) |
| GO:0019538 | protein metabolic process | 4.09E-05 | 1.56E-03 | 1076/3656 (29.4%) | 4028/14979 (26.8%) |
| GO:0006073 | cellular glucan metabolic process | 1.11E-04 | 4.05E-03 | 53/3656 (1.4%) | 136/14979 (0.9%) |
| GO:0044042 | glucan metabolic process | 1.38E-04 | 4.85E-03 | 53/3656 (1.4%) | 137/14979 (0.9%) |
| GO:0015074 | DNA integration | 1.58E-04 | 5.33E-03 | 62/3656 (1.6%) | 167/14979 (1.1%) |
| GO:0005976 | polysaccharide metabolic process | 1.81E-04 | 5.88E-03 | 69/3656 (1.8%) | 191/14979 (1.2%) |
| GO:0006032 | chitin catabolic process | 2.25E-04 | 6.80E-03 | 11/3656 (0.3%) | 16/14979 (0.1%) |
| GO:0006026 | aminoglycan catabolic process | 2.25E-04 | 6.80E-03 | 11/3656 (0.3%) | 16/14979 (0.1%) |
| GO:0006278 | RNA-dependent DNA replication | 2.57E-04 | 7.52E-03 | 69/3656 (1.8%) | 193/14979 (1.2%) |
| GO:0042401 | cellular biogenic amine biosynthetic process | 2.75E-04 | 7.77E-03 | 10/3656 (0.2%) | 14/14979 (0.0%) |
| GO:0044238 | primary metabolic process | 3.31E-04 | 9.07E-03 | 1860/3656 (50.8%) | 7252/14979 (48.4%) |
| GO:0071554 | cell wall organization or biogenesis | 3.93E-04 | 1.04E-02 | 53/3656 (1.4%) | 142/14979 (0.9%) |
| GO:0005992 | trehalose biosynthetic process | 6.06E-04 | 1.56E-02 | 20/3656 (0.5%) | 41/14979 (0.2%) |
| GO:0005991 | trehalose metabolic process | 9.33E-04 | 2.27E-02 | 21/3656 (0.5%) | 45/14979 (0.3%) |
| GO:0051258 | protein polymerization | 9.41E-04 | 2.27E-02 | 29/3656 (0.7%) | 69/14979 (0.4%) |
| GO:0016998 | cell wall macromolecule catabolic process | 9.80E-04 | 2.27E-02 | 23/3656 (0.6%) | 51/14979 (0.3%) |
| GO:0006030 | chitin metabolic process | 9.96E-04 | 2.27E-02 | 11/3656 (0.3%) | 18/14979 (0.1%) |
| GO:0044264 | cellular polysaccharide metabolic process | 1.01E-03 | 2.27E-02 | 53/3656 (1.4%) | 147/14979 (0.9%) |
| GO:0016138 | glycoside biosynthetic process | 1.29E-03 | 2.76E-02 | 20/3656 (0.5%) | 43/14979 (0.2%) |
| GO:0046351 | disaccharide biosynthetic process | 1.29E-03 | 2.76E-02 | 20/3656 (0.5%) | 43/14979 (0.2%) |
| GO:0009312 | oligosaccharide biosynthetic process | 1.33E-03 | 2.77E-02 | 21/3656 (0.5%) | 46/14979 (0.3%) |
| GO:0065007 | biological regulation | 2.00E-03 | 4.08E-02 | 565/3656 (15.4%) | 2096/14979 (13.9%) |
| GO:0044036 | cell wall macromolecule metabolic process | 2.44E-03 | 4.86E-02 | 24/3656 (0.6%) | 57/14979 (0.3%) |

**Table S13: Gene Ontology Terms Enriched in *A. deserti* Leaf Cluster B**

| **goterm** | **name** | **p-value** | **corrected_p-value** | **Cluster Frequency** | **Total Frequency** |
| --- | --- | --- | --- | --- | --- |
| GO:0006464 | cellular protein modification process | 6.97E-16 | 5.79E-13 | 401/2242 (17.8%) | 1872/14979 (12.4%) |
| GO:0043412 | macromolecule modification | 1.20E-14 | 4.97E-12 | 406/2242 (18.1%) | 1933/14979 (12.9%) |
| GO:0043687 | post-translational protein modification | 4.64E-14 | 1.28E-11 | 360/2242 (16.0%) | 1685/14979 (11.2%) |
| GO:0006468 | protein phosphorylation | 9.73E-13 | 2.02E-10 | 323/2242 (14.4%) | 1509/14979 (10.0%) |
| GO:0030244 | cellulose biosynthetic process | 1.62E-11 | 1.99E-09 | 37/2242 (1.6%) | 79/14979 (0.5%) |
| GO:0033692 | cellular polysaccharide biosynthetic process | 1.65E-11 | 1.99E-09 | 45/2242 (2.0%) | 108/14979 (0.7%) |
| GO:0009250 | glucan biosynthetic process | 1.74E-11 | 1.99E-09 | 42/2242 (1.8%) | 97/14979 (0.6%) |
| GO:0000271 | polysaccharide biosynthetic process | 1.92E-11 | 1.99E-09 | 47/2242 (2.0%) | 116/14979 (0.7%) |
| GO:0030243 | cellulose metabolic process | 2.59E-11 | 2.39E-09 | 37/2242 (1.6%) | 80/14979 (0.5%) |
| GO:0006796 | phosphate-containing compound metabolic process | 3.83E-11 | 2.89E-09 | 343/2242 (15.2%) | 1668/14979 (11.1%) |
| GO:0006793 | phosphorus metabolic process | 3.83E-11 | 2.89E-09 | 343/2242 (15.2%) | 1668/14979 (11.1%) |
| GO:0016310 | phosphorylation | 1.25E-10 | 8.66E-09 | 330/2242 (14.7%) | 1608/14979 (10.7%) |
| GO:0016051 | carbohydrate biosynthetic process | 1.20E-08 | 7.66E-07 | 58/2242 (2.5%) | 185/14979 (1.2%) |
| GO:0005976 | polysaccharide metabolic process | 1.65E-08 | 9.80E-07 | 59/2242 (2.6%) | 191/14979 (1.2%) |
| GO:0044264 | cellular polysaccharide metabolic process | 1.81E-08 | 1.00E-06 | 49/2242 (2.1%) | 147/14979 (0.9%) |
| GO:0034637 | cellular carbohydrate biosynthetic process | 2.07E-08 | 1.07E-06 | 55/2242 (2.4%) | 174/14979 (1.1%) |
| GO:0006073 | cellular glucan metabolic process | 2.96E-08 | 1.44E-06 | 46/2242 (2.0%) | 136/14979 (0.9%) |
| GO:0044042 | glucan metabolic process | 3.81E-08 | 1.76E-06 | 46/2242 (2.0%) | 137/14979 (0.9%) |
| GO:0070882 | cellular cell wall organization or biogenesis | 7.15E-08 | 3.12E-06 | 16/2242 (0.7%) | 26/14979 (0.1%) |
| GO:0007047 | cellular cell wall organization | 1.24E-07 | 4.91E-06 | 12/2242 (0.5%) | 16/14979 (0.1%) |
| GO:0045229 | external encapsulating structure organization | 1.24E-07 | 4.91E-06 | 12/2242 (0.5%) | 16/14979 (0.1%) |
| GO:0044262 | cellular carbohydrate metabolic process | 6.98E-06 | 2.63E-04 | 121/2242 (5.3%) | 554/14979 (3.6%) |
| GO:0009415 | response to water | 9.62E-06 | 3.47E-04 | 12/2242 (0.5%) | 21/14979 (0.1%) |
| GO:0006486 | protein glycosylation | 2.30E-05 | 6.83E-04 | 26/2242 (1.1%) | 76/14979 (0.5%) |
| GO:0009101 | glycoprotein biosynthetic process | 2.30E-05 | 6.83E-04 | 26/2242 (1.1%) | 76/14979 (0.5%) |
| GO:0009100 | glycoprotein metabolic process | 2.30E-05 | 6.83E-04 | 26/2242 (1.1%) | 76/14979 (0.5%) |
| GO:0043413 | macromolecule glycosylation | 2.30E-05 | 6.83E-04 | 26/2242 (1.1%) | 76/14979 (0.5%) |
| GO:0070085 | glycosylation | 2.30E-05 | 6.83E-04 | 26/2242 (1.1%) | 76/14979 (0.5%) |
| GO:0044260 | cellular macromolecule metabolic process | 7.10E-05 | 2.03E-03 | 781/2242 (34.8%) | 4696/14979 (31.3%) |
| GO:0005975 | carbohydrate metabolic process | 1.56E-04 | 4.32E-03 | 212/2242 (9.4%) | 1128/14979 (7.5%) |
| GO:0008643 | carbohydrate transport | 2.38E-04 | 6.38E-03 | 6/2242 (0.2%) | 8/14979 (0.0%) |
| GO:0009628 | response to abiotic stimulus | 4.24E-04 | 1.10E-02 | 23/2242 (1.0%) | 75/14979 (0.5%) |
| GO:0043170 | macromolecule metabolic process | 8.30E-04 | 2.09E-02 | 864/2242 (38.5%) | 5328/14979 (35.5%) |
| GO:0044267 | cellular protein metabolic process | 1.56E-03 | 3.82E-02 | 574/2242 (25.6%) | 3465/14979 (23.1%) |
| GO:0032012 | regulation of ARF protein signal transduction | 1.91E-03 | 4.53E-02 | 13/2242 (0.5%) | 37/14979 (0.2%) |

**Table S14: Gene Ontology Terms Enriched in *A. deserti* Leaf Cluster C**

| **goterm** | **name** | **p-value** | **corrected_p-value** | **Cluster Frequency** | **Total Frequency** |
| --- | --- | --- | --- | --- | --- |
| GO:0016192 | vesicle-mediated transport | 3.43E-20 | 2.83E-17 | 98/2071 (4.7%) | 275/14979 (1.8%) |
| GO:0051649 | establishment of localization in cell | 2.12E-17 | 8.73E-15 | 116/2071 (5.6%) | 382/14979 (2.5%) |
| GO:0051641 | cellular localization | 6.56E-16 | 1.80E-13 | 119/2071 (5.7%) | 413/14979 (2.7%) |
| GO:0006886 | intracellular protein transport | 1.32E-14 | 2.73E-12 | 87/2071 (4.2%) | 274/14979 (1.8%) |
| GO:0015031 | protein transport | 3.71E-14 | 5.10E-12 | 102/2071 (4.9%) | 350/14979 (2.3%) |
| GO:0045184 | establishment of protein localization | 3.71E-14 | 5.10E-12 | 102/2071 (4.9%) | 350/14979 (2.3%) |
| GO:0046907 | intracellular transport | 1.59E-13 | 1.78E-11 | 96/2071 (4.6%) | 328/14979 (2.1%) |
| GO:0034613 | cellular protein localization | 1.94E-13 | 1.78E-11 | 90/2071 (4.3%) | 300/14979 (2.0%) |
| GO:0070727 | cellular macromolecule localization | 1.94E-13 | 1.78E-11 | 90/2071 (4.3%) | 300/14979 (2.0%) |
| GO:0008104 | protein localization | 2.67E-13 | 2.20E-11 | 107/2071 (5.1%) | 385/14979 (2.5%) |
| GO:0033036 | macromolecule localization | 4.47E-13 | 3.35E-11 | 110/2071 (5.3%) | 403/14979 (2.6%) |
| GO:0006464 | cellular protein modification process | 2.13E-11 | 1.46E-09 | 355/2071 (17.1%) | 1872/14979 (12.4%) |
| GO:0043412 | macromolecule modification | 2.86E-11 | 1.82E-09 | 364/2071 (17.5%) | 1933/14979 (12.9%) |
| GO:0006810 | transport | 1.67E-09 | 9.18E-08 | 339/2071 (16.3%) | 1835/14979 (12.2%) |
| GO:0051234 | establishment of localization | 1.67E-09 | 9.18E-08 | 339/2071 (16.3%) | 1835/14979 (12.2%) |
| GO:0051179 | localization | 2.04E-09 | 1.05E-07 | 344/2071 (16.6%) | 1870/14979 (12.4%) |
| GO:0006468 | protein phosphorylation | 7.20E-09 | 3.49E-07 | 284/2071 (13.7%) | 1509/14979 (10.0%) |
| GO:0043687 | post-translational protein modification | 3.34E-08 | 1.53E-06 | 308/2071 (14.8%) | 1685/14979 (11.2%) |
| GO:0016310 | phosphorylation | 2.24E-07 | 9.73E-06 | 291/2071 (14.0%) | 1608/14979 (10.7%) |
| GO:0006796 | phosphate-containing compound metabolic process | 3.67E-07 | 1.44E-05 | 299/2071 (14.4%) | 1668/14979 (11.1%) |
| GO:0006793 | phosphorus metabolic process | 3.67E-07 | 1.44E-05 | 299/2071 (14.4%) | 1668/14979 (11.1%) |
| GO:0022406 | membrane docking | 5.32E-06 | 1.91E-04 | 14/2071 (0.6%) | 28/14979 (0.1%) |
| GO:0048278 | vesicle docking | 5.32E-06 | 1.91E-04 | 14/2071 (0.6%) | 28/14979 (0.1%) |
| GO:0046578 | regulation of Ras protein signal transduction | 6.05E-06 | 2.08E-04 | 25/2071 (1.2%) | 72/14979 (0.4%) |
| GO:0010646 | regulation of cell communication | 1.05E-05 | 2.99E-04 | 27/2071 (1.3%) | 83/14979 (0.5%) |
| GO:0009966 | regulation of signal transduction | 1.05E-05 | 2.99E-04 | 27/2071 (1.3%) | 83/14979 (0.5%) |
| GO:0023051 | regulation of signaling | 1.05E-05 | 2.99E-04 | 27/2071 (1.3%) | 83/14979 (0.5%) |
| GO:0051056 | regulation of small GTPase mediated signal transduction | 1.05E-05 | 2.99E-04 | 25/2071 (1.2%) | 74/14979 (0.4%) |
| GO:0035466 | regulation of signal transduction | 1.05E-05 | 2.99E-04 | 25/2071 (1.2%) | 74/14979 (0.4%) |
| GO:0032940 | secretion by cell | 1.71E-05 | 4.40E-04 | 20/2071 (0.9%) | 54/14979 (0.3%) |
| GO:0006887 | exocytosis | 1.71E-05 | 4.40E-04 | 20/2071 (0.9%) | 54/14979 (0.3%) |
| GO:0046903 | secretion | 1.71E-05 | 4.40E-04 | 20/2071 (0.9%) | 54/14979 (0.3%) |
| GO:0009987 | cellular process | 2.00E-05 | 4.99E-04 | 1226/2071 (59.1%) | 8241/14979 (55.0%) |
| GO:0044265 | cellular macromolecule catabolic process | 3.62E-05 | 8.78E-04 | 55/2071 (2.6%) | 233/14979 (1.5%) |
| GO:0033124 | regulation of GTP catabolic process | 5.75E-05 | 1.22E-03 | 22/2071 (1.0%) | 67/14979 (0.4%) |
| GO:0033121 | regulation of purine nucleotide catabolic process | 5.75E-05 | 1.22E-03 | 22/2071 (1.0%) | 67/14979 (0.4%) |
| GO:0032318 | regulation of Ras GTPase activity | 5.75E-05 | 1.22E-03 | 22/2071 (1.0%) | 67/14979 (0.4%) |
| GO:0043087 | regulation of GTPase activity | 5.75E-05 | 1.22E-03 | 22/2071 (1.0%) | 67/14979 (0.4%) |
| GO:0030811 | regulation of nucleotide catabolic process | 5.75E-05 | 1.22E-03 | 22/2071 (1.0%) | 67/14979 (0.4%) |
| GO:0051336 | regulation of hydrolase activity | 6.41E-05 | 1.32E-03 | 23/2071 (1.1%) | 72/14979 (0.4%) |
| GO:0051603 | proteolysis involved in cellular protein catabolic process | 8.19E-05 | 1.61E-03 | 52/2071 (2.5%) | 223/14979 (1.4%) |
| GO:0044257 | cellular protein catabolic process | 8.19E-05 | 1.61E-03 | 52/2071 (2.5%) | 223/14979 (1.4%) |
| GO:0030163 | protein catabolic process | 1.05E-04 | 2.01E-03 | 52/2071 (2.5%) | 225/14979 (1.5%) |
| GO:0006511 | ubiquitin-dependent protein catabolic process | 1.24E-04 | 2.32E-03 | 43/2071 (2.0%) | 177/14979 (1.1%) |
| GO:0043632 | modification-dependent macromolecule catabolic process | 1.42E-04 | 2.54E-03 | 43/2071 (2.0%) | 178/14979 (1.1%) |
| GO:0019941 | modification-dependent protein catabolic process | 1.42E-04 | 2.54E-03 | 43/2071 (2.0%) | 178/14979 (1.1%) |
| GO:0006140 | regulation of nucleotide metabolic process | 1.61E-04 | 2.83E-03 | 23/2071 (1.1%) | 76/14979 (0.5%) |
| GO:0006261 | DNA-dependent DNA replication | 1.89E-04 | 3.21E-03 | 7/2071 (0.3%) | 11/14979 (0.0%) |
| GO:0031329 | regulation of cellular catabolic process | 1.90E-04 | 3.21E-03 | 22/2071 (1.0%) | 72/14979 (0.4%) |
| GO:0009894 | regulation of catabolic process | 2.47E-04 | 4.08E-03 | 23/2071 (1.1%) | 78/14979 (0.5%) |
| GO:0032501 | multicellular organismal process | 3.04E-04 | 4.92E-03 | 23/2071 (1.1%) | 79/14979 (0.5%) |
| GO:0009057 | macromolecule catabolic process | 3.48E-04 | 5.53E-03 | 58/2071 (2.8%) | 270/14979 (1.8%) |
| GO:0006904 | vesicle docking involved in exocytosis | 5.11E-04 | 7.95E-03 | 10/2071 (0.4%) | 23/14979 (0.1%) |
| GO:0032012 | regulation of ARF protein signal transduction | 9.05E-04 | 1.38E-02 | 13/2071 (0.6%) | 37/14979 (0.2%) |
| GO:0006486 | protein glycosylation | 1.16E-03 | 1.62E-02 | 21/2071 (1.0%) | 76/14979 (0.5%) |
| GO:0009101 | glycoprotein biosynthetic process | 1.16E-03 | 1.62E-02 | 21/2071 (1.0%) | 76/14979 (0.5%) |
| GO:0009100 | glycoprotein metabolic process | 1.16E-03 | 1.62E-02 | 21/2071 (1.0%) | 76/14979 (0.5%) |
| GO:0043413 | macromolecule glycosylation | 1.16E-03 | 1.62E-02 | 21/2071 (1.0%) | 76/14979 (0.5%) |
| GO:0070085 | glycosylation | 1.16E-03 | 1.62E-02 | 21/2071 (1.0%) | 76/14979 (0.5%) |
| GO:0050790 | regulation of catalytic activity | 1.28E-03 | 1.77E-02 | 38/2071 (1.8%) | 168/14979 (1.1%) |
| GO:0065009 | regulation of molecular function | 1.44E-03 | 1.95E-02 | 38/2071 (1.8%) | 169/14979 (1.1%) |
| GO:0046488 | phosphatidylinositol metabolic process | 1.58E-03 | 2.09E-02 | 8/2071 (0.3%) | 18/14979 (0.1%) |
| GO:0006269 | DNA replication, synthesis of RNA primer | 1.62E-03 | 2.09E-02 | 4/2071 (0.1%) | 5/14979 (0.0%) |
| GO:0006891 | intra-Golgi vesicle-mediated transport | 1.62E-03 | 2.09E-02 | 4/2071 (0.1%) | 5/14979 (0.0%) |
| GO:0032313 | regulation of Rab GTPase activity | 1.80E-03 | 2.25E-02 | 12/2071 (0.5%) | 35/14979 (0.2%) |
| GO:0032483 | regulation of Rab protein signal transduction | 1.80E-03 | 2.25E-02 | 12/2071 (0.5%) | 35/14979 (0.2%) |
| GO:0032502 | developmental process | 2.23E-03 | 2.71E-02 | 10/2071 (0.4%) | 27/14979 (0.1%) |
| GO:0007275 | multicellular organismal development | 2.23E-03 | 2.71E-02 | 10/2071 (0.4%) | 27/14979 (0.1%) |

**Table S15: Gene Ontology Terms Enriched in *A. deserti* Leaf Cluster D**

| **goterm** | **name** | **p-value** | **corrected_p-value** | **Cluster Frequency** | **Total Frequency** |
| --- | --- | --- | --- | --- | --- |
| GO:0006259 | DNA metabolic process | 6.07E-10 | 4.03E-07 | 71/833 (8.5%) | 592/14979 (3.9%) |
| GO:0090304 | nucleic acid metabolic process | 1.34E-09 | 4.46E-07 | 117/833 (14.0%) | 1208/14979 (8.0%) |
| GO:0006139 | nucleobase-containing compound metabolic process | 1.60E-07 | 3.54E-05 | 132/833 (15.8%) | 1534/14979 (10.2%) |
| GO:0006278 | RNA-dependent DNA replication | 2.96E-07 | 4.91E-05 | 30/833 (3.6%) | 193/14979 (1.2%) |
| GO:0015074 | DNA integration | 1.75E-06 | 2.33E-04 | 26/833 (3.1%) | 167/14979 (1.1%) |
| GO:0022904 | respiratory electron transport chain | 2.99E-06 | 3.31E-04 | 9/833 (1.0%) | 24/14979 (0.1%) |
| GO:0006807 | nitrogen compound metabolic process | 5.54E-06 | 5.25E-04 | 153/833 (18.3%) | 1961/14979 (13.0%) |
| GO:0034641 | cellular nitrogen compound metabolic process | 6.52E-06 | 5.41E-04 | 148/833 (17.7%) | 1889/14979 (12.6%) |
| GO:0022900 | electron transport chain | 1.04E-05 | 7.64E-04 | 10/833 (1.2%) | 34/14979 (0.2%) |
| GO:0006260 | DNA replication | 2.38E-05 | 1.58E-03 | 33/833 (3.9%) | 274/14979 (1.8%) |
| GO:0042773 | ATP synthesis coupled electron transport | 4.48E-05 | 2.70E-03 | 7/833 (0.8%) | 19/14979 (0.1%) |
| GO:0043933 | macromolecular complex subunit organization | 1.58E-04 | 8.73E-03 | 27/833 (3.2%) | 227/14979 (1.5%) |
| GO:0065003 | macromolecular complex assembly | 3.34E-04 | 1.70E-02 | 25/833 (3.0%) | 213/14979 (1.4%) |
| GO:0022607 | cellular component assembly | 3.59E-04 | 1.70E-02 | 25/833 (3.0%) | 214/14979 (1.4%) |
| GO:0045333 | cellular respiration | 4.13E-04 | 1.83E-02 | 9/833 (1.0%) | 42/14979 (0.2%) |
| GO:0015980 | energy derivation by oxidation of organic compounds | 5.94E-04 | 2.47E-02 | 9/833 (1.0%) | 44/14979 (0.2%) |
| GO:0008535 | respiratory chain complex IV assembly | 6.57E-04 | 2.57E-02 | 3/833 (0.3%) | 4/14979 (0.0%) |
| GO:0034621 | cellular macromolecular complex subunit organization | 9.87E-04 | 3.64E-02 | 19/833 (2.2%) | 155/14979 (1.0%) |
| GO:0044085 | cellular component biogenesis | 1.16E-03 | 4.05E-02 | 30/833 (3.6%) | 297/14979 (1.9%) |
| GO:0009896 | positive regulation of catabolic process | 1.57E-03 | 4.55E-02 | 3/833 (0.3%) | 5/14979 (0.0%) |
| GO:0031331 | positive regulation of cellular catabolic process | 1.57E-03 | 4.55E-02 | 3/833 (0.3%) | 5/14979 (0.0%) |
| GO:0010506 | regulation of autophagy | 1.57E-03 | 4.55E-02 | 3/833 (0.3%) | 5/14979 (0.0%) |
| GO:0010508 | positive regulation of autophagy | 1.57E-03 | 4.55E-02 | 3/833 (0.3%) | 5/14979 (0.0%) |

**Table S16: Gene Ontology Terms Enriched in *A. deserti* Leaf Cluster E**

| **goterm** | **name** | **p-value** | **corrected_p-value** | **Cluster Frequency** | **Total Frequency** |
| --- | --- | --- | --- | --- | --- |
| GO:0015979 | photosynthesis | 1.35E-39 | 1.29E-36 | 81/3682 (2.1%) | 90/14979 (0.6%) |
| GO:0015672 | monovalent inorganic cation transport | 2.95E-11 | 1.28E-08 | 94/3682 (2.5%) | 206/14979 (1.3%) |
| GO:0006818 | hydrogen transport | 5.37E-11 | 1.28E-08 | 83/3682 (2.2%) | 176/14979 (1.1%) |
| GO:0015992 | proton transport | 5.37E-11 | 1.28E-08 | 83/3682 (2.2%) | 176/14979 (1.1%) |
| GO:0034220 | ion transmembrane transport | 7.05E-11 | 1.35E-08 | 71/3682 (1.9%) | 143/14979 (0.9%) |
| GO:0006508 | proteolysis | 2.72E-10 | 4.33E-08 | 259/3682 (7.0%) | 751/14979 (5.0%) |
| GO:0008152 | metabolic process | 5.29E-10 | 7.23E-08 | 2758/3682 (74.9%) | 10630/14979 (70.9%) |
| GO:0015988 | energy coupled proton transport, against electrochemical gradient | 2.56E-09 | 2.72E-07 | 48/3682 (1.3%) | 89/14979 (0.5%) |
| GO:0015991 | ATP hydrolysis coupled proton transport | 2.56E-09 | 2.72E-07 | 48/3682 (1.3%) | 89/14979 (0.5%) |
| GO:0033013 | tetrapyrrole metabolic process | 2.57E-07 | 2.41E-05 | 29/3682 (0.7%) | 49/14979 (0.3%) |
| GO:0051188 | cofactor biosynthetic process | 2.77E-07 | 2.41E-05 | 53/3682 (1.4%) | 114/14979 (0.7%) |
| GO:0051186 | cofactor metabolic process | 3.87E-07 | 3.09E-05 | 76/3682 (2.0%) | 184/14979 (1.2%) |
| GO:0018130 | heterocycle biosynthetic process | 4.51E-07 | 3.32E-05 | 43/3682 (1.1%) | 87/14979 (0.5%) |
| GO:0006778 | porphyrin-containing compound metabolic process | 5.77E-07 | 3.95E-05 | 20/3682 (0.5%) | 29/14979 (0.1%) |
| GO:0006812 | cation transport | 7.16E-07 | 4.57E-05 | 144/3682 (3.9%) | 409/14979 (2.7%) |
| GO:0046483 | heterocycle metabolic process | 1.91E-06 | 1.14E-04 | 117/3682 (3.1%) | 324/14979 (2.1%) |
| GO:0006352 | DNA-dependent transcription, initiation | 4.56E-06 | 2.57E-04 | 33/3682 (0.8%) | 65/14979 (0.4%) |
| GO:0033014 | tetrapyrrole biosynthetic process | 4.85E-06 | 2.58E-04 | 25/3682 (0.6%) | 44/14979 (0.2%) |
| GO:0006811 | ion transport | 5.50E-06 | 2.77E-04 | 156/3682 (4.2%) | 464/14979 (3.0%) |
| GO:0043039 | tRNA aminoacylation | 6.60E-06 | 2.84E-04 | 48/3682 (1.3%) | 109/14979 (0.7%) |
| GO:0043038 | amino acid activation | 6.60E-06 | 2.84E-04 | 48/3682 (1.3%) | 109/14979 (0.7%) |
| GO:0006721 | terpenoid metabolic process | 6.82E-06 | 2.84E-04 | 10/3682 (0.2%) | 11/14979 (0.0%) |
| GO:0016114 | terpenoid biosynthetic process | 6.82E-06 | 2.84E-04 | 10/3682 (0.2%) | 11/14979 (0.0%) |
| GO:0006418 | tRNA aminoacylation for protein translation | 1.20E-05 | 4.77E-04 | 45/3682 (1.2%) | 102/14979 (0.6%) |
| GO:0006779 | porphyrin-containing compound biosynthetic process | 1.58E-05 | 6.05E-04 | 16/3682 (0.4%) | 24/14979 (0.1%) |
| GO:0042440 | pigment metabolic process | 1.96E-05 | 7.20E-04 | 15/3682 (0.4%) | 22/14979 (0.1%) |
| GO:0006399 | tRNA metabolic process | 3.26E-05 | 1.14E-03 | 65/3682 (1.7%) | 168/14979 (1.1%) |
| GO:0019748 | secondary metabolic process | 3.32E-05 | 1.14E-03 | 11/3682 (0.2%) | 14/14979 (0.0%) |
| GO:0044281 | small molecule metabolic process | 3.80E-05 | 1.25E-03 | 364/3682 (9.8%) | 1241/14979 (8.2%) |
| GO:0055114 | oxidation-reduction process | 5.58E-05 | 1.78E-03 | 527/3682 (14.3%) | 1865/14979 (12.4%) |
| GO:0008299 | isoprenoid biosynthetic process | 9.14E-05 | 2.73E-03 | 19/3682 (0.5%) | 34/14979 (0.2%) |
| GO:0006720 | isoprenoid metabolic process | 9.14E-05 | 2.73E-03 | 19/3682 (0.5%) | 34/14979 (0.2%) |
| GO:0005996 | monosaccharide metabolic process | 1.17E-04 | 3.40E-03 | 71/3682 (1.9%) | 194/14979 (1.2%) |
| GO:0006066 | alcohol metabolic process | 1.59E-04 | 4.48E-03 | 88/3682 (2.3%) | 253/14979 (1.6%) |
| GO:0009260 | ribonucleotide biosynthetic process | 1.78E-04 | 4.87E-03 | 35/3682 (0.9%) | 81/14979 (0.5%) |
| GO:0009201 | ribonucleoside triphosphate biosynthetic process | 2.27E-04 | 5.57E-03 | 28/3682 (0.7%) | 61/14979 (0.4%) |
| GO:0009206 | purine ribonucleoside triphosphate biosynthetic process | 2.27E-04 | 5.57E-03 | 28/3682 (0.7%) | 61/14979 (0.4%) |
| GO:0009145 | purine nucleoside triphosphate biosynthetic process | 2.27E-04 | 5.57E-03 | 28/3682 (0.7%) | 61/14979 (0.4%) |
| GO:0009142 | nucleoside triphosphate biosynthetic process | 2.27E-04 | 5.57E-03 | 28/3682 (0.7%) | 61/14979 (0.4%) |
| GO:0009152 | purine ribonucleotide biosynthetic process | 2.41E-04 | 5.77E-03 | 33/3682 (0.8%) | 76/14979 (0.5%) |
| GO:0034660 | ncRNA metabolic process | 2.78E-04 | 6.49E-03 | 74/3682 (2.0%) | 209/14979 (1.3%) |
| GO:0006007 | glucose catabolic process | 3.12E-04 | 6.78E-03 | 57/3682 (1.5%) | 153/14979 (1.0%) |
| GO:0046365 | monosaccharide catabolic process | 3.12E-04 | 6.78E-03 | 57/3682 (1.5%) | 153/14979 (1.0%) |
| GO:0019320 | hexose catabolic process | 3.12E-04 | 6.78E-03 | 57/3682 (1.5%) | 153/14979 (1.0%) |
| GO:0019318 | hexose metabolic process | 3.61E-04 | 7.68E-03 | 67/3682 (1.8%) | 187/14979 (1.2%) |
| GO:0006091 | generation of precursor metabolites and energy | 3.73E-04 | 7.76E-03 | 77/3682 (2.0%) | 221/14979 (1.4%) |
| GO:0006164 | purine nucleotide biosynthetic process | 3.94E-04 | 8.02E-03 | 37/3682 (1.0%) | 90/14979 (0.6%) |
| GO:0016070 | RNA metabolic process | 4.02E-04 | 8.02E-03 | 191/3682 (5.1%) | 628/14979 (4.1%) |
| GO:0006096 | glycolysis | 4.26E-04 | 8.32E-03 | 47/3682 (1.2%) | 122/14979 (0.8%) |
| GO:0009056 | catabolic process | 4.80E-04 | 9.18E-03 | 179/3682 (4.8%) | 586/14979 (3.9%) |
| GO:0046148 | pigment biosynthetic process | 5.30E-04 | 9.95E-03 | 11/3682 (0.2%) | 17/14979 (0.1%) |
| GO:0006511 | ubiquitin-dependent protein catabolic process | 6.49E-04 | 1.19E-02 | 63/3682 (1.7%) | 177/14979 (1.1%) |
| GO:0008617 | guanosine metabolic process | 6.83E-04 | 1.20E-02 | 10/3682 (0.2%) | 15/14979 (0.1%) |
| GO:0030163 | protein catabolic process | 6.85E-04 | 1.20E-02 | 77/3682 (2.0%) | 225/14979 (1.5%) |
| GO:0006006 | glucose metabolic process | 6.90E-04 | 1.20E-02 | 62/3682 (1.6%) | 174/14979 (1.1%) |
| GO:0009119 | ribonucleoside metabolic process | 7.48E-04 | 1.24E-02 | 22/3682 (0.5%) | 47/14979 (0.3%) |
| GO:0044271 | cellular nitrogen compound biosynthetic process | 7.50E-04 | 1.24E-02 | 122/3682 (3.3%) | 384/14979 (2.5%) |
| GO:0043632 | modification-dependent macromolecule catabolic process | 7.67E-04 | 1.24E-02 | 63/3682 (1.7%) | 178/14979 (1.1%) |
| GO:0019941 | modification-dependent protein catabolic process | 7.67E-04 | 1.24E-02 | 63/3682 (1.7%) | 178/14979 (1.1%) |
| GO:0045454 | cell redox homeostasis | 9.25E-04 | 1.48E-02 | 60/3682 (1.6%) | 169/14979 (1.1%) |
| GO:0006754 | ATP biosynthetic process | 1.06E-03 | 1.67E-02 | 22/3682 (0.5%) | 48/14979 (0.3%) |
| GO:0046164 | alcohol catabolic process | 1.24E-03 | 1.91E-02 | 58/3682 (1.5%) | 164/14979 (1.0%) |
| GO:0051603 | proteolysis involved in cellular protein catabolic process | 1.38E-03 | 2.04E-02 | 75/3682 (2.0%) | 223/14979 (1.4%) |
| GO:0044257 | cellular protein catabolic process | 1.38E-03 | 2.04E-02 | 75/3682 (2.0%) | 223/14979 (1.4%) |
| GO:0009165 | nucleotide biosynthetic process | 1.40E-03 | 2.04E-02 | 44/3682 (1.1%) | 118/14979 (0.7%) |
| GO:0034654 | nucleobase-containing compound biosynthetic process | 1.43E-03 | 2.04E-02 | 47/3682 (1.2%) | 128/14979 (0.8%) |
| GO:0034404 | nucleobase-containing small molecule biosynthetic process | 1.43E-03 | 2.04E-02 | 47/3682 (1.2%) | 128/14979 (0.8%) |
| GO:0044275 | cellular carbohydrate catabolic process | 1.46E-03 | 2.05E-02 | 58/3682 (1.5%) | 165/14979 (1.1%) |
| GO:0042592 | homeostatic process | 1.55E-03 | 2.15E-02 | 62/3682 (1.6%) | 179/14979 (1.1%) |
| GO:0019725 | cellular homeostasis | 1.65E-03 | 2.26E-02 | 61/3682 (1.6%) | 176/14979 (1.1%) |
| GO:0015969 | guanosine tetraphosphate metabolic process | 2.47E-03 | 3.32E-02 | 8/3682 (0.2%) | 12/14979 (0.0%) |
| GO:0043467 | regulation of generation of precursor metabolites and energy | 3.65E-03 | 4.65E-02 | 4/3682 (0.1%) | 4/14979 (0.0%) |
| GO:0042549 | photosystem II stabilization | 3.65E-03 | 4.65E-02 | 4/3682 (0.1%) | 4/14979 (0.0%) |
| GO:0042548 | regulation of photosynthesis, light reaction | 3.65E-03 | 4.65E-02 | 4/3682 (0.1%) | 4/14979 (0.0%) |
| GO:0010109 | regulation of photosynthesis | 3.65E-03 | 4.65E-02 | 4/3682 (0.1%) | 4/14979 (0.0%) |
| GO:0046173 | polyol biosynthetic process | 3.84E-03 | 4.78E-02 | 6/3682 (0.1%) | 8/14979 (0.0%) |
| GO:0006021 | inositol biosynthetic process | 3.84E-03 | 4.78E-02 | 6/3682 (0.1%) | 8/14979 (0.0%) |
| GO:0046034 | ATP metabolic process | 4.10E-03 | 4.99E-02 | 27/3682 (0.7%) | 68/14979 (0.4%) |
| GO:0016117 | carotenoid biosynthetic process | 4.27E-03 | 4.99E-02 | 5/3682 (0.1%) | 6/14979 (0.0%) |
| GO:0016116 | carotenoid metabolic process | 4.27E-03 | 4.99E-02 | 5/3682 (0.1%) | 6/14979 (0.0%) |
| GO:0016108 | tetraterpenoid metabolic process | 4.27E-03 | 4.99E-02 | 5/3682 (0.1%) | 6/14979 (0.0%) |
| GO:0016109 | tetraterpenoid biosynthetic process | 4.27E-03 | 4.99E-02 | 5/3682 (0.1%) | 6/14979 (0.0%) |

**Table S17: Gene Ontology Terms Enriched in *A. deserti* Leaf Cluster F**

| **goterm** | **name** | **p-value** | **corrected_p-value** | **Cluster Frequency** | **Total Frequency** |
| --- | --- | --- | --- | --- | --- |
| GO:0010468 | regulation of gene expression | 2.93E-07 | 8.98E-05 | 150/1088 (13.7%) | 1389/14979 (9.2%) |
| GO:0060255 | regulation of macromolecule metabolic process | 3.56E-07 | 8.98E-05 | 151/1088 (13.8%) | 1405/14979 (9.3%) |
| GO:0009889 | regulation of biosynthetic process | 6.39E-07 | 8.98E-05 | 148/1088 (13.6%) | 1384/14979 (9.2%) |
| GO:0031326 | regulation of cellular biosynthetic process | 6.39E-07 | 8.98E-05 | 148/1088 (13.6%) | 1384/14979 (9.2%) |
| GO:0010556 | regulation of macromolecule biosynthetic process | 6.39E-07 | 8.98E-05 | 148/1088 (13.6%) | 1384/14979 (9.2%) |
| GO:0051252 | regulation of RNA metabolic process | 2.94E-06 | 2.69E-04 | 140/1088 (12.8%) | 1328/14979 (8.8%) |
| GO:0006355 | regulation of transcription, DNA-dependent | 2.94E-06 | 2.69E-04 | 140/1088 (12.8%) | 1328/14979 (8.8%) |
| GO:0045449 | regulation of transcription, DNA-dependent | 3.07E-06 | 2.69E-04 | 140/1088 (12.8%) | 1329/14979 (8.8%) |
| GO:0080090 | regulation of primary metabolic process | 6.95E-06 | 5.42E-04 | 150/1088 (13.7%) | 1467/14979 (9.7%) |
| GO:0019222 | regulation of metabolic process | 8.02E-06 | 5.63E-04 | 155/1088 (14.2%) | 1530/14979 (10.2%) |
| GO:0031323 | regulation of cellular metabolic process | 1.40E-05 | 8.97E-04 | 152/1088 (13.9%) | 1510/14979 (10.0%) |
| GO:0051171 | regulation of nitrogen compound metabolic process | 2.14E-05 | 1.16E-03 | 143/1088 (13.1%) | 1415/14979 (9.4%) |
| GO:0019219 | regulation of nucleobase-containing compound metabolic process | 2.14E-05 | 1.16E-03 | 143/1088 (13.1%) | 1415/14979 (9.4%) |
| GO:0050789 | regulation of biological process | 4.25E-05 | 2.13E-03 | 189/1088 (17.3%) | 1992/14979 (13.2%) |
| GO:0065007 | biological regulation | 9.90E-05 | 4.63E-03 | 195/1088 (17.9%) | 2096/14979 (13.9%) |
| GO:0050794 | regulation of cellular process | 1.40E-04 | 6.14E-03 | 183/1088 (16.8%) | 1960/14979 (13.0%) |

**Table S18: High confidence proteins composing core pathways of CAM photosynthesis**

|  |  |  | ***Agave tequilana*** | |  | ***Agave deserti*** | |
| --- | --- | --- | --- | --- | --- | --- | --- |
| **KEGG Orthology Number** | **KEGG description** | **Role** | **No. high-confidence proteins** | **protein list** |  | **No. high-confidence proteins** | **protein list** |
| K01595 | phosphoenolpyruvate carboxylase (PEPC) | CAM - dark | 11 | Locus1448v1rpkm157.97_16, Locus17657v1rpkm15.39_19, Locus2509v1rpkm105.37_7, Locus2723v1rpkm98.64_12, Locus29350v1rpkm6.28_2, Locus33636v1rpkm4.48_2, **Locus346v1rpkm398.98_17**, Locus38408v1rpkm3.10_3, Locus4211v1rpkm67.72_6, Locus44884v1rpkm2.04_2, Locus7190v1rpkm41.57_7 |  | 11 | Locus12291v1rpkm22.33_7, Locus2544v1rpkm116.26_9, Locus3392v1rpkm88.29_13, Locus34012v1rpkm3.32_2, Locus4288v1rpkm70.34_18, Locus5233v1rpkm58.01_11, Locus5245v1rpkm57.94_4, **Locus59v1rpkm1185.23_16**, Locus7058v1rpkm42.73_6, Locus8641v1rpkm34.28_17, Locus8921v1rpkm33.15_3 |
| K00026 | malate dehydrogenase | CAM - dark | 12 | Locus102553v1rpkm0.63_4, Locus107859v1rpkm0.60_6, Locus16029v1rpkm17.46_7, Locus1899v1rpkm129.88_7, Locus22979v1rpkm10.33_4, Locus2540v1rpkm104.20_8, Locus3329v1rpkm82.87_8, Locus3508v1rpkm79.33_7, Locus4052v1rpkm70.02_7, Locus52613v1rpkm1.43_6, Locus57892v1rpkm1.22_7, Locus90250v1rpkm0.72_2 |  | 14 | Locus3789v1rpkm79.17_10, Locus11380v1rpkm24.50_4, Locus14695v1rpkm17.70_6, Locus16095v1rpkm15.55_2, Locus27458v1rpkm5.91_5, Locus28122v1rpkm5.58_2, Locus39844v1rpkm1.99_8, Locus43032v1rpkm1.54_5, Locus4501v1rpkm67.17_8, Locus59196v1rpkm0.64_3, Locus73766v1rpkm0.43_1, Locus7467v1rpkm40.33_8, Locus9331v1rpkm31.53_6, Locus9658v1rpkm30.15_7 |
| K00025 | malate dehydrogenase | CAM - dark | 2 | Locus25427v1rpkm8.52_6, Locus831v1rpkm232.62_6 |  | 3 | Locus20361v1rpkm10.70_6, Locus2679v1rpkm110.76_7, Locus783v1rpkm290.40_6 |
| K00029 | malate dehydrogenase | CAM - light | 4 | Locus1050v1rpkm200.15_6, Locus2290v1rpkm113.06_6, Locus3060v1rpkm88.68_6, Locus7119v1rpkm41.97_4 |  | 5 | Locus10276v1rpkm28.01_1, Locus1104v1rpkm225.15_3, Locus1989v1rpkm143.06_9, Locus40682v1rpkm1.85_3, Locus42162v1rpkm1.64_5 |
| K01006 | pyruvate dikinase (PPDK) | CAM - light | 5 | Locus1302v1rpkm171.11_10, Locus21709v1rpkm11.32_2, Locus25752v1rpkm8.30_7, Locus4130v1rpkm68.89_3, Locus4453v1rpkm64.48_2 |  | 2 | Locus19v1rpkm2035.53_4, Locus221v1rpkm662.77_3 |

Presumptive photosynthetic isoforms of PEPC based on highest expression in leaves are in bold.

**Figure S1:**


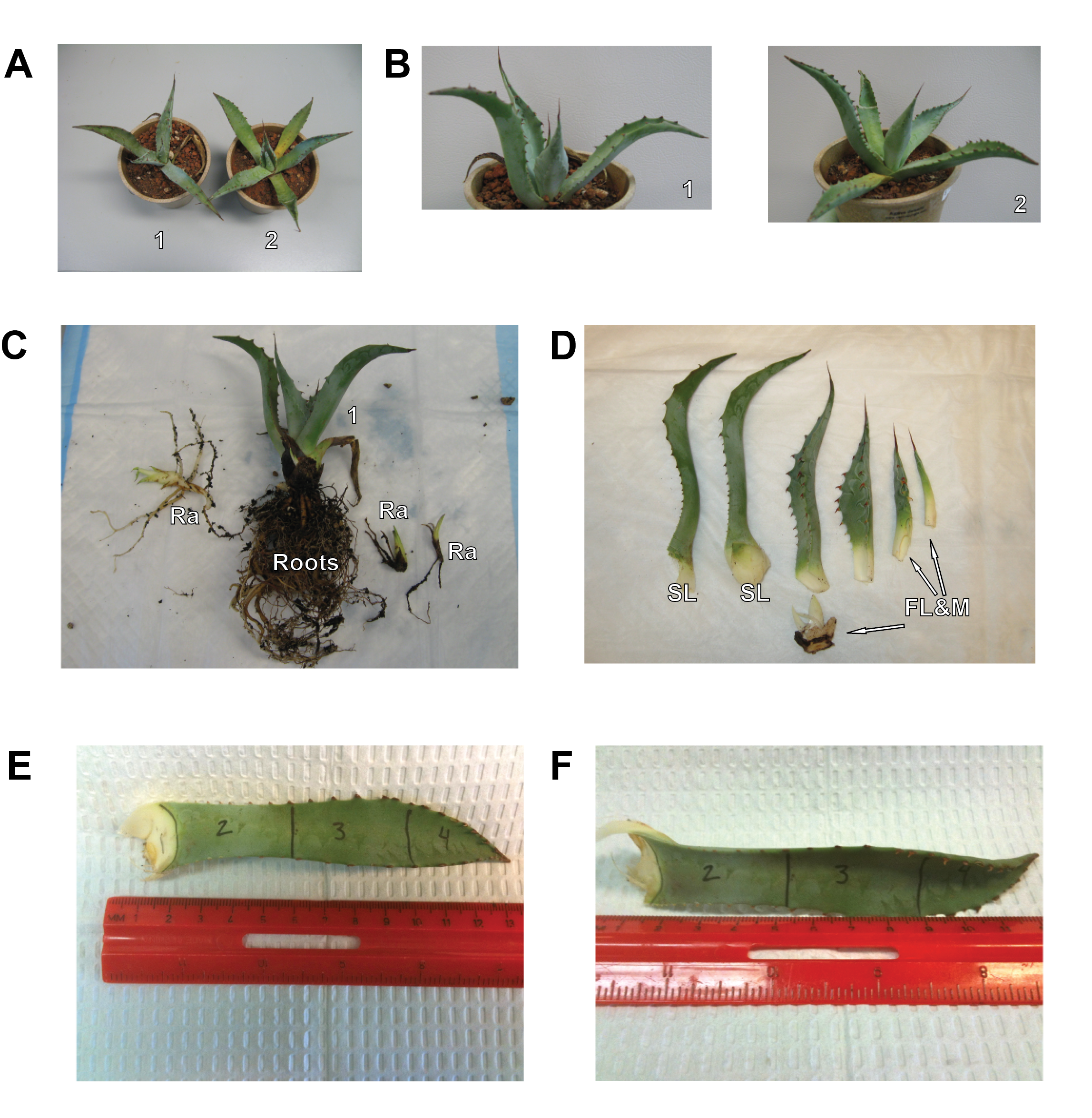


**Figure S1.** *Agave* *deserti* plants used for transcriptome assembly and expression analysis. (A) *A. deserti* sibling plants #1 and #2, top view. (B) Sibling plants #1 and #2, lateral view. (C) Individual #1 extricated from soil, displaying ramets (Ra) and roots used for RNA-seq samples. (D) Dissected portions of individual #2. SL—samples leaves for proximal-distal analyses. FL&M—folded leaves and meristematic tissue. (E, F) Close-up of the two leaves sampled for the proximal-distal leaf analysis.

**Figure S2:**


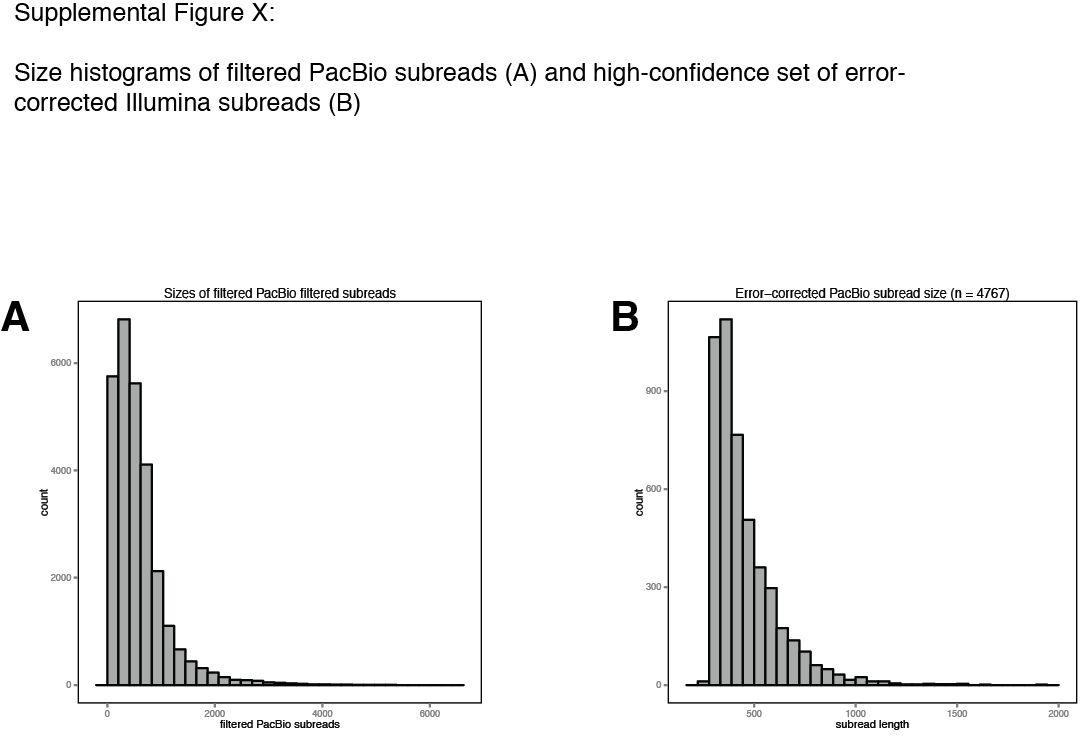


**Figure S2.** Histograms of *A. tequilana* PacBio subread lengths. (A) Uncorrected subread lengths. (B) Subread lengths of remaining high-confidence error-corrected PacBio subreads.

**Figure S3:**


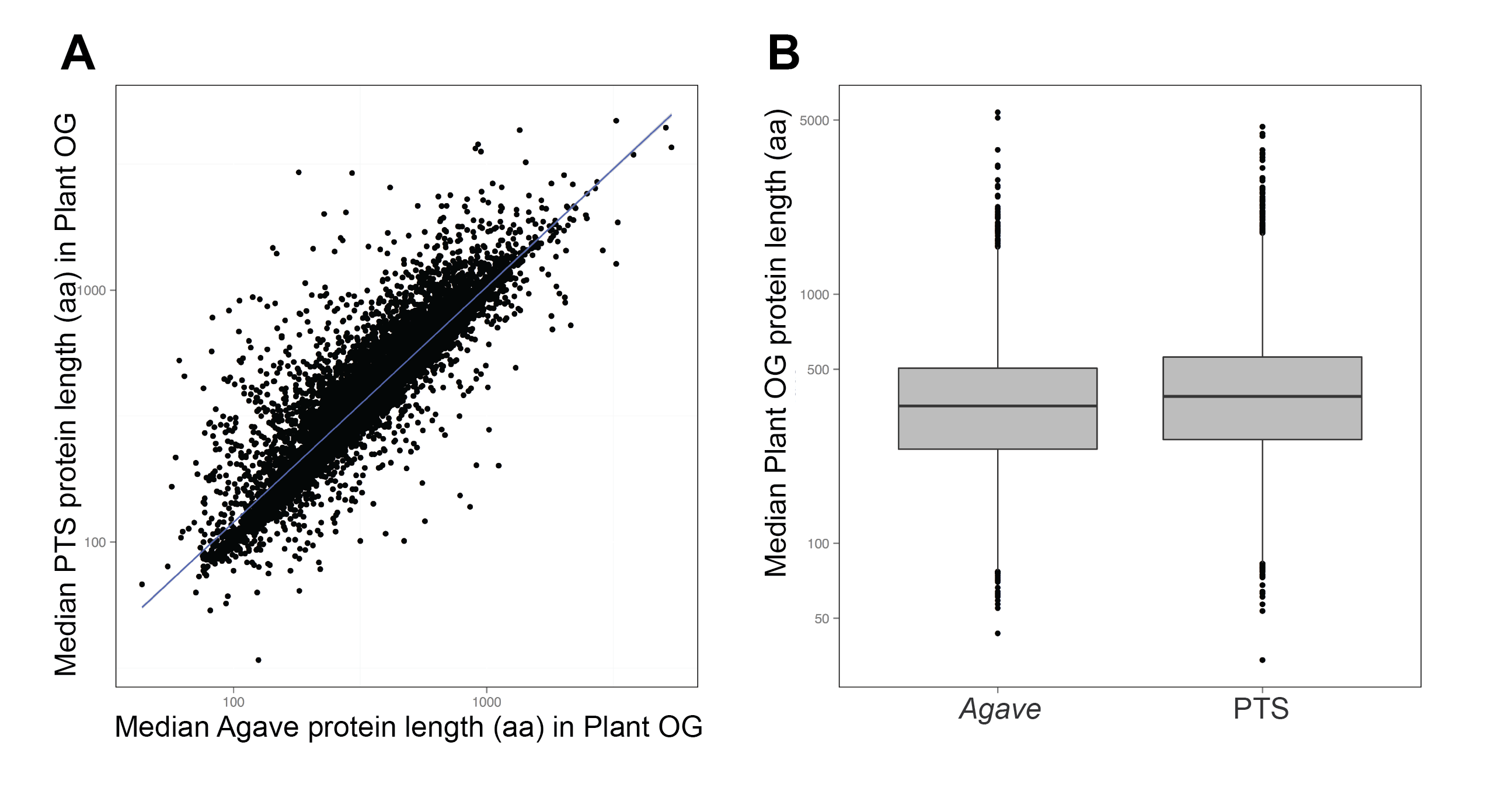


**Figure S3.** *Agave* protein lengths are similar to those in other plant species. (A) Scatterplot of median protein lengths in 12,346 Plant OGs for *Agave* and the PTS. Blue line represents best-fit linear model for (slope = 0.9942, y-intercept = 50.6 amino acids). Correlation is 0.85. (B) Boxplots of median Plant OG lengths for *Agave* (median = 356 aa) and the PTS (median = 389 aa).

**Figure S4:**


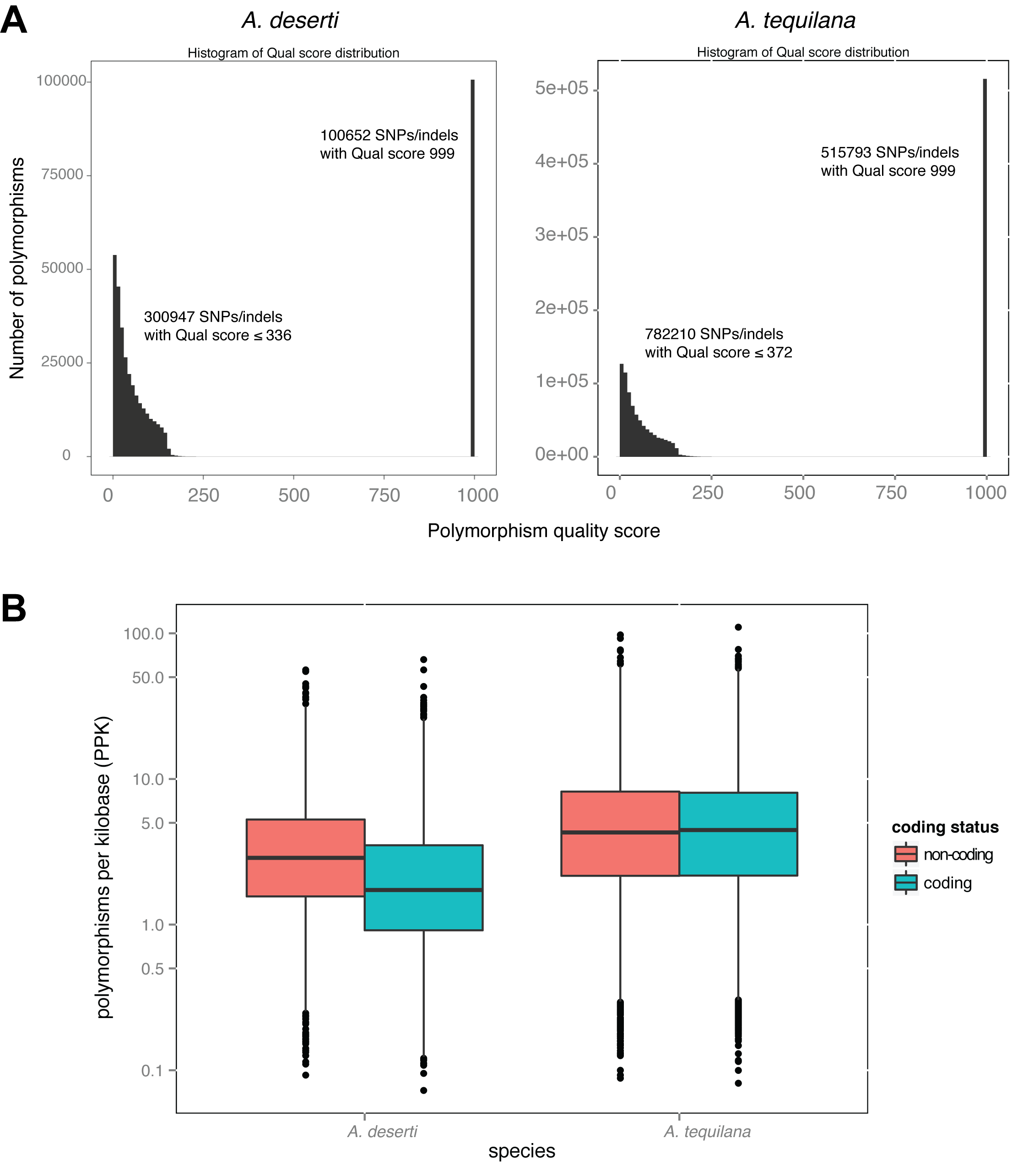


**Figure S4.** Detection of polymorphisms in the *A. deserti* reference transcriptome. (A) Histogram of quality values of SNP and indels detected by SAMtools by aligning all reads to the v1 transcript *Agave* assemblies, demonstrating a bimodal distribution. Lower quality SNPs and indels (quality score < 999) represent a mixture of poorly matching reads spanning splice junctions and various SNPs and indels not present in the v1 reference set and sequencing errors. (B) Boxplots of the number of polymorphisms per kilobase (PPK) for coding and non-coding sequences. Coding vs. non-coding PPK for *A. deserti* is significant (Wilcoxon Rank Sum test *p*-value < 0.05).

**Figure S5:**

**
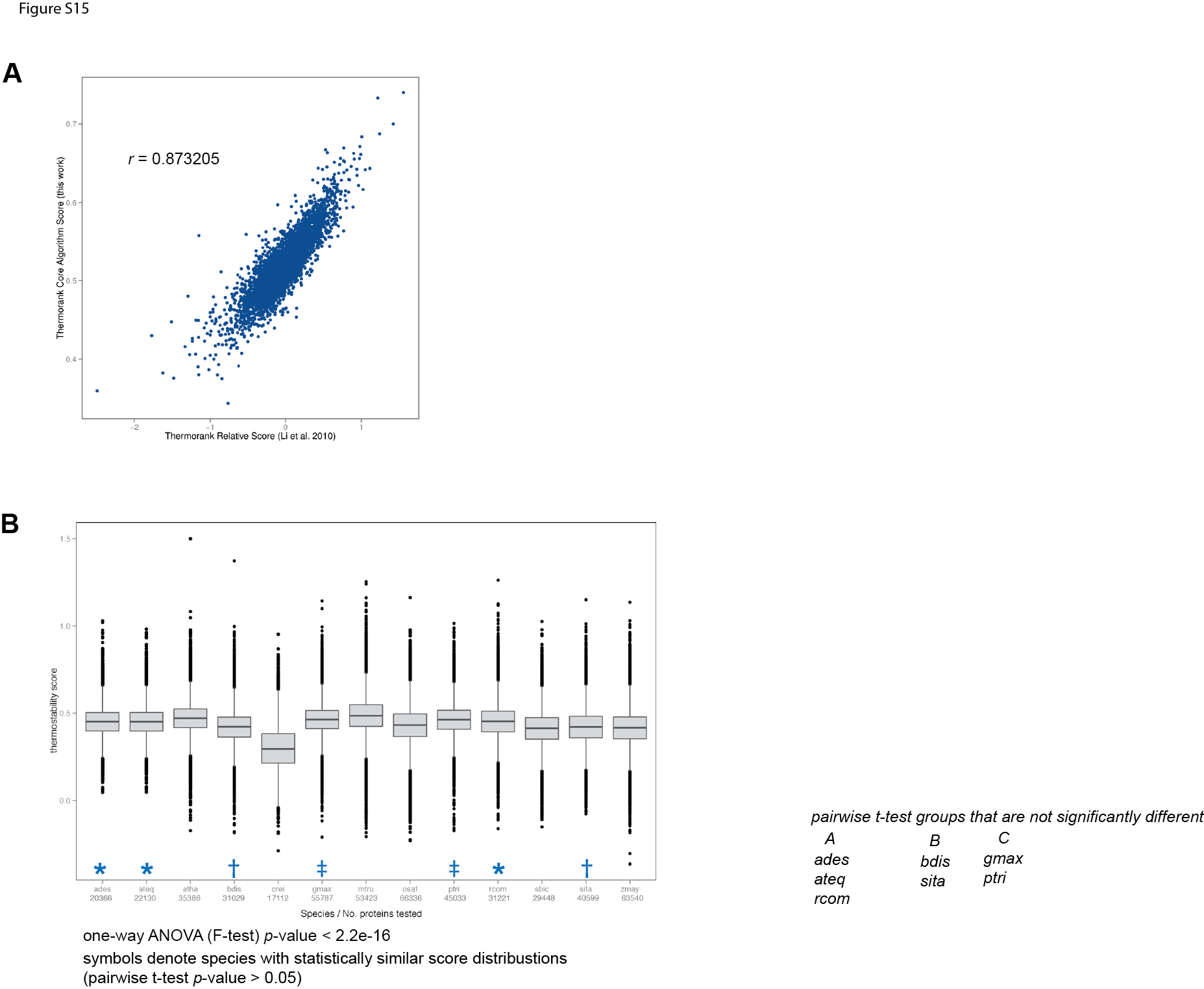
**

**Figure S5**. Analysis of inferred plant proteomes using a modified version of Thermorank [1]. (A) Correlation of Thermorank scores with a modified version capable of using large datasets. (B) Boxplots of the thermostability scores for agaves and the additional 11 species it the Phytozome Tester Set. Symbols (*, †, ‡) denote species groups with statistically similar overall thermostability scores (pairwise *t*-test *p* > 0.05). Species abbreviations: (ades—*A. deserti*, ateq—*A. tequilana*, atha—*Arabidopsis thaliana*, bdis—*Brachypodium distachyon*, crei—*Chlamydomonas reinhardtii*, gmax—*Glycine max*, mtru—*Medicago truncatula*, osat—*Oryza sativa*, ptri—*Populus trichocarpa*, rcom—*Ricinus communis*, sbic—*Sorghum bicolor*, sita—*Setaria italica*, zmay—*Zea mays*. Numbers below species name indicate total number of proteins in each species tested.

**Figure S6:**


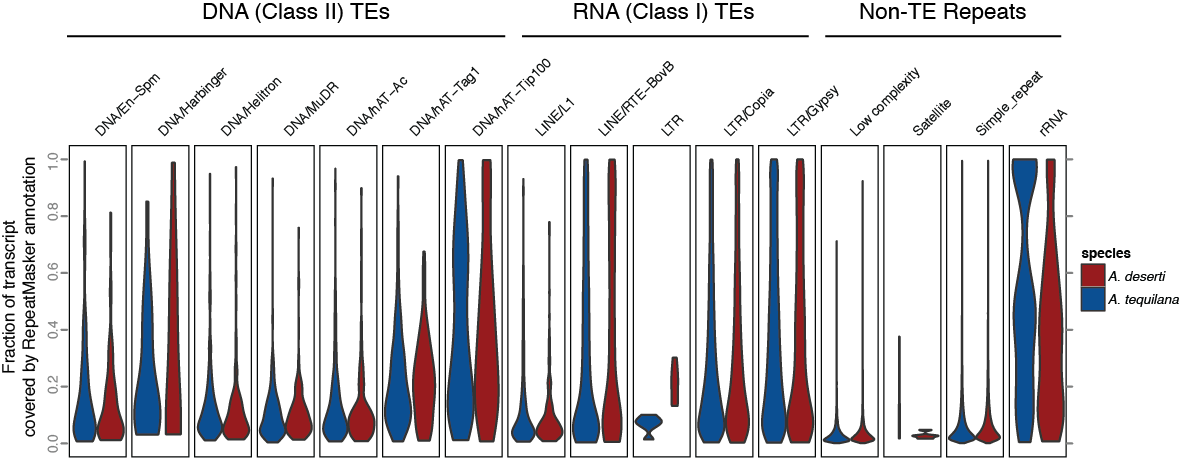


**Figure S6.** Violin plots of the fraction of transcript coverage by indicated RepeatMasker annotations.

**Figure S7:**


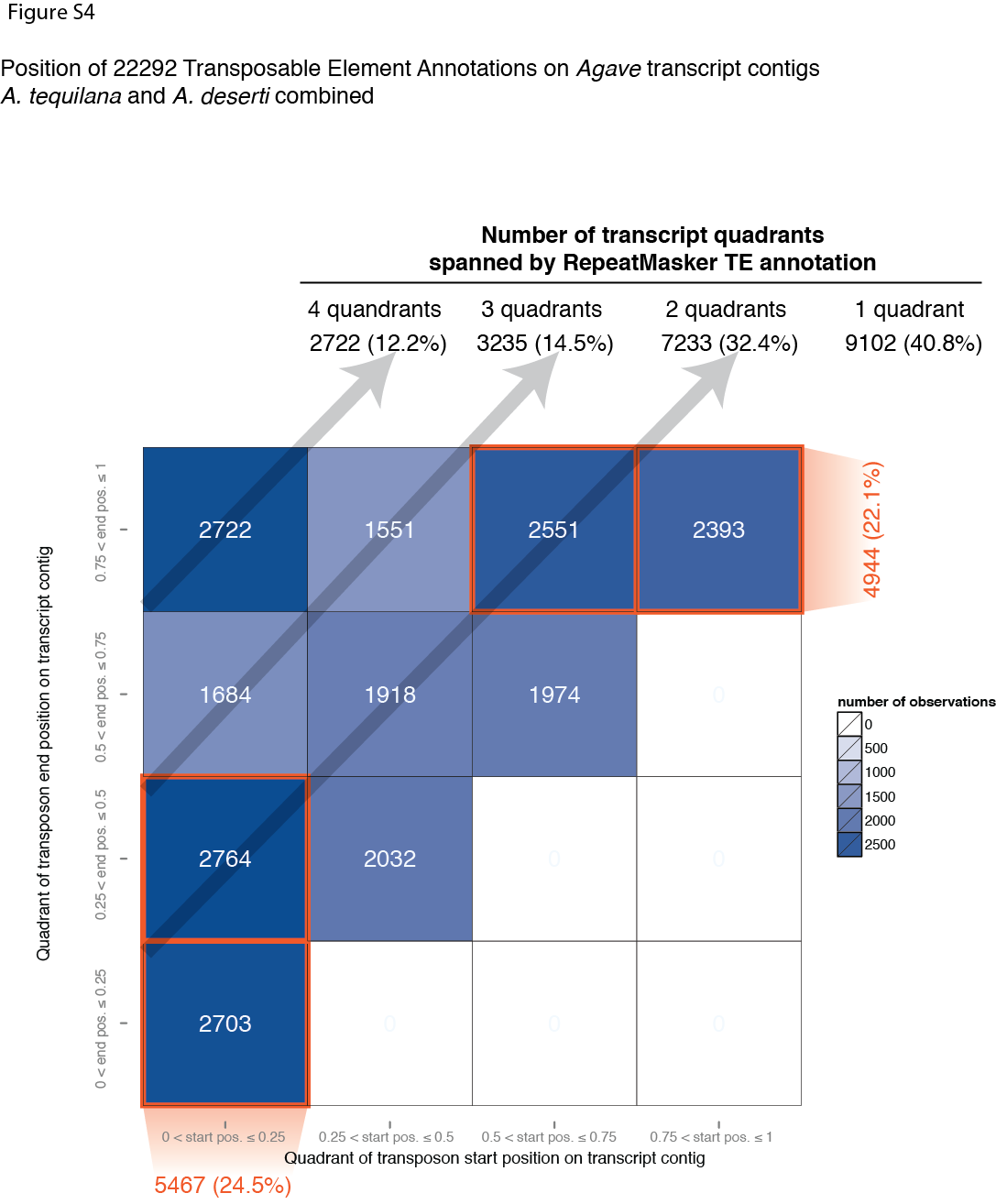


**Figure S7.** Position of RepeatMasker transposable element annotations on 22292 *A. tequilana* and *A. deserti* transcripts (combined datasets). Transcripts were divided into 4 quadrants. The numbers indicate the total number of transcripts with annotations starting (x-axis) and ending (y-axis) in various quadrants. Boxes outlined in orange indicate the number of annotations starting and terminating in solely 5’ or 3’ regions of the transcript, indicating transcription initiation or termination in transposable element sequences.

**Figure S8:**


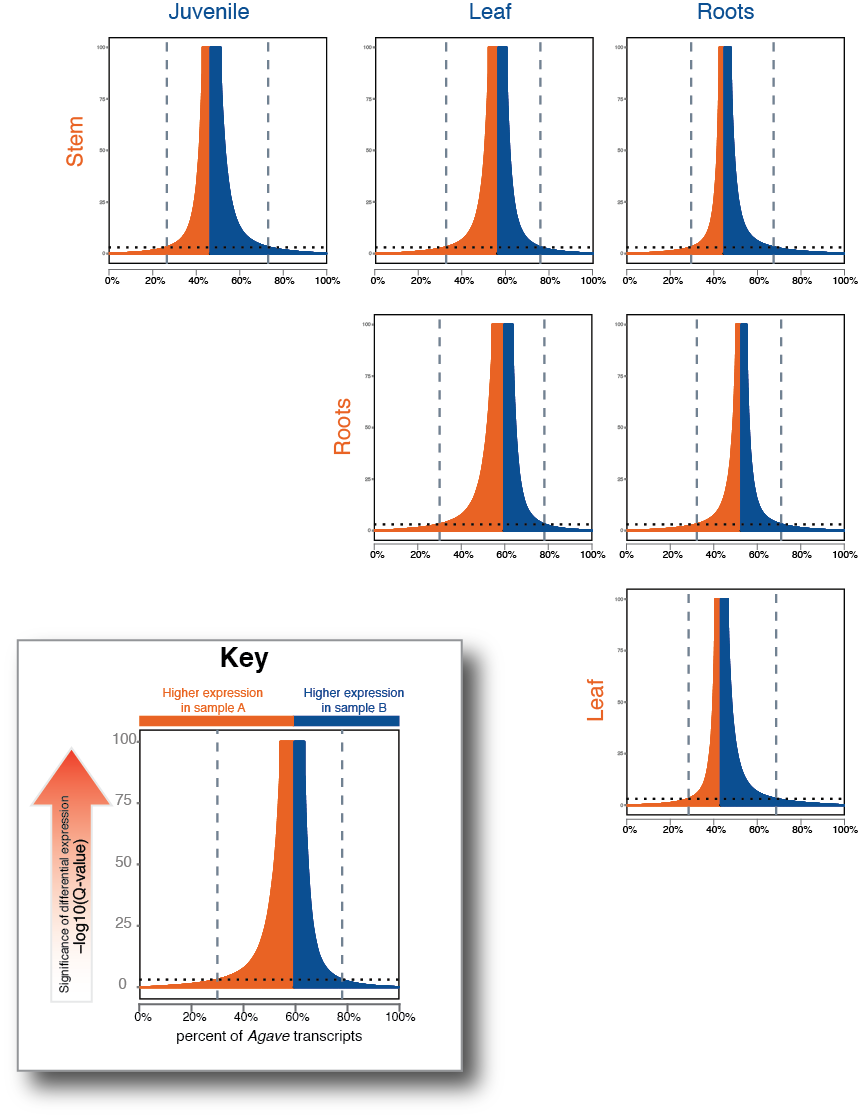


**Figure S8.** Differential expression plots between *A. tequilana* tissues. Inset: key. Y-axis is the negative log_10_ (Q-value), where higher numbers reflect more significantly distinct expression values, x-axis represent individual transcripts ordered by Q-value and sample. Dotted and dashed lines represents a Q-value cutoff 0.001. Total width of peak is proportional to the number of differentially expressed transcripts, colored orange if more abundant in Sample A, blue if more abundant in Sample B.

**Figure S9:**


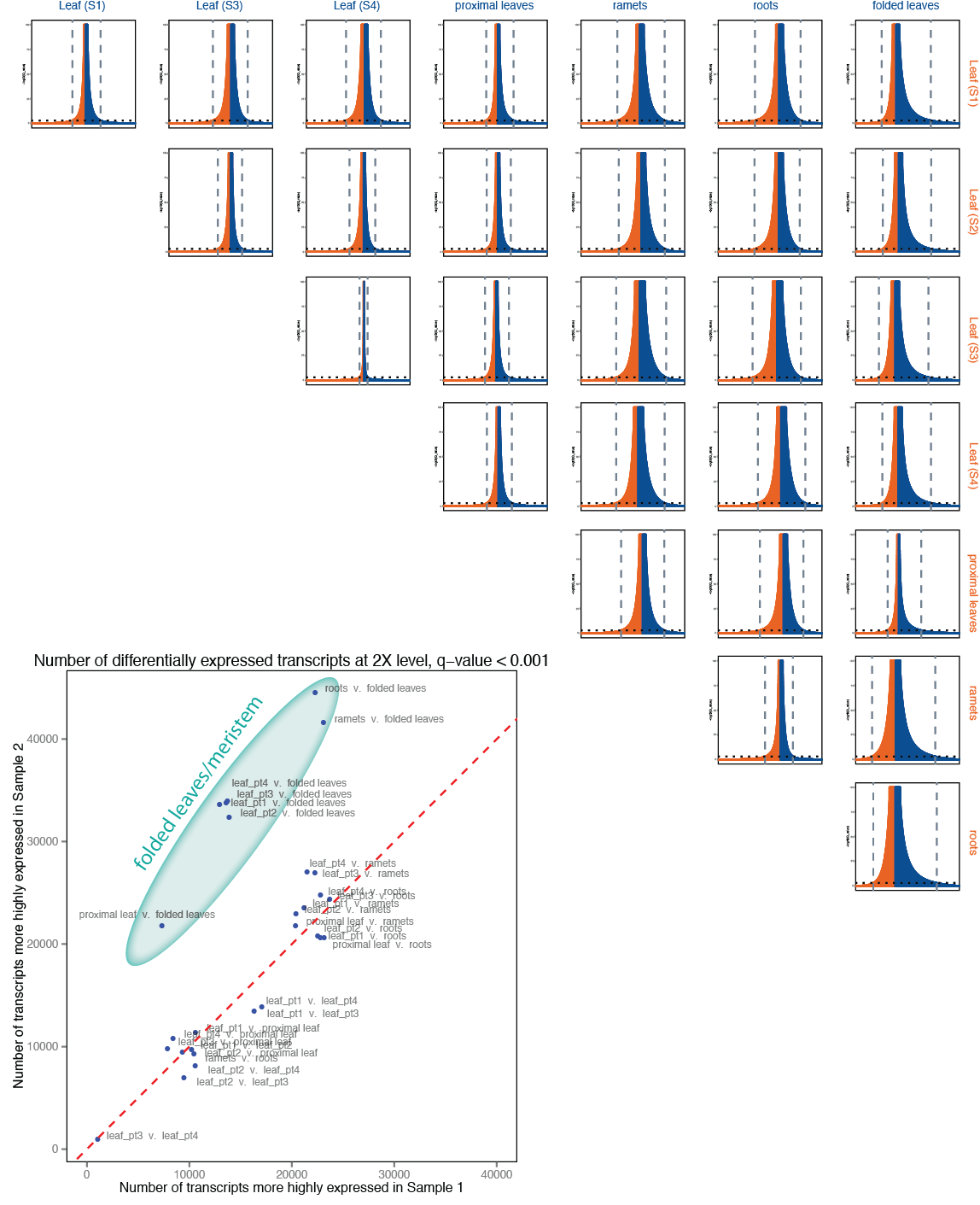


**Figure S9.** Differential expression plots between *A. deserti* tissues. Plots are as described in Figure S8. Inset—scatterplot of the number of differentially expressed transcripts between tissues. Cyan ellipse denotes sample comparisons with skewed distribution of more abundant transcripts in the meristem and folded leaf sample.

**Figure S10:**


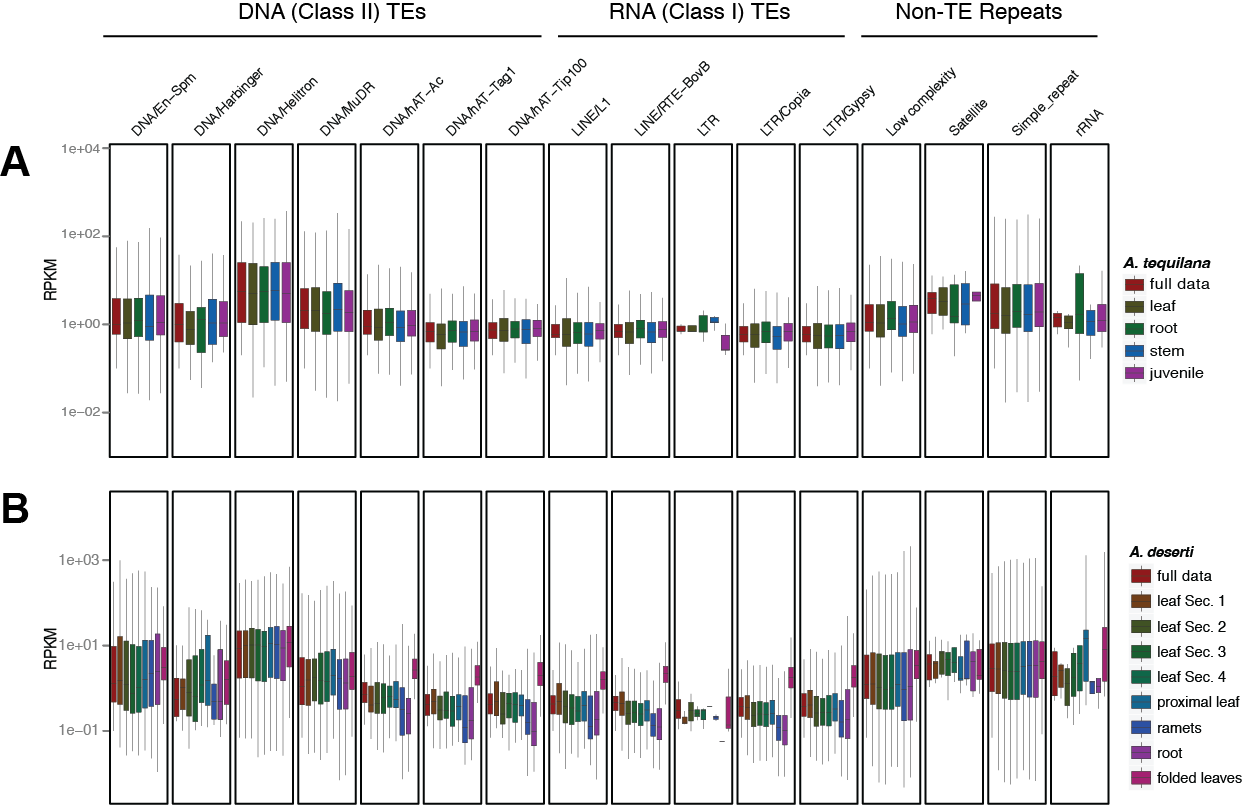


**Figure S10.** Expression of transposable elements in agave tissues. (A) *A. tequilana* and (B) *A. deserti*. “Full data” expression refers to RPKM value as provided immediately after initial assembly by Rnnotator. Note significantly higher expression of some transposable elements in the *A. deserti* folded leaves/meristem sample.

**References**

1. Li Y, Middaugh CR, Fang J: **A novel scoring function for discriminating hyperthermophilic and mesophilic proteins with application to predicting relative thermostability of protein mutants**. *BMC Bioinformatics* 2010, **11**:62.
